# An Antagonistic Photovoltaic Memristor for Bioinspired Active Contrast Adaptation

**DOI:** 10.1002/adma.202409844

**Published:** 2024-10-30

**Authors:** Guodong Gong, You Zhou, Ziyu Xiong, Tao Sun, Huaxin Li, Qingxiu Li, Wenyu Zhao, Guohua Zhang, Yongbiao Zhai, Ziyu Lv, Hongwei Tan, Ye Zhou, Su‐Ting Han

**Affiliations:** ^1^ Institute of Microscale Optoelectronics Shenzhen University Shenzhen 518060 P. R. China; ^2^ College of Electronics and Information Engineering Shenzhen University Shenzhen 518060 P. R. China; ^3^ Institute of Polymer Optoelectronic Materials and Devices State Key Laboratory of Luminescent Materials and Devices South China University of Technology Guangzhou 510640 P. R. China; ^4^ Key Laboratory of Physics and Technology for Advanced Batteries (Ministry of Education) College of Physics Jilin University Changchun 130012 P. R. China; ^5^ NanoSpin Department of Applied Physics Aalto University School of Science P.O. Box 15100 Aalto FI‐00076 Finland; ^6^ Institute for Advanced Study Shenzhen University Shenzhen 518060 P. R. China; ^7^ Department of Applied Biology and Chemical Technology The Hong Kong Polytechnic University Hung Hom Hong Kong Kowloon 999077 P. R. China

**Keywords:** active contrast adaptation, antagonistic photovoltaics, bioinspired electronics, photodoping, self‐powered memristor

## Abstract

Machine vision systems that consist of cameras and image‐processing components for visual inspection and identification tasks play a critical role in various intelligent applications, including pilotless vehicles and surveillance systems. However, current systems usually possess a limited dynamic range and fixed photoresponsivity, restricting their capability of gaining high‐fidelity images when encoding a high‐contrast scene. Here, it is shown that a photovoltaic memristor incorporating two antagonistic photovoltaic junctions can autonomously adjust its response to varying light stimuli, enabling the amplification of shadows and inhibition of highlight saturation. Due to the dynamic photodoping effect at the p‐n junction with an asymmetrical profile, the photocurrent polarities of the antagonistic memristor can be changed as the light intensity increases. The light‐intensity‐dependent switchable photovoltaic behaviors match Weber's law where photosensitivity is inversely proportional to the light stimuli. An 11 × 11 memristor array is used to detect a high‐contrast scene with light intensities ranging from 1 to 5 × 10^4^ µW cm^−2^, achieving a similar active contrast adaptation performance compared with the human visual systems (less than 1.2 s at 94 dB). This work paves the way for innovative neuromorphic device designs and may lead to the development of state‐of‐the‐art active visual adaptation photosensors.

## Graphical abstract

A bioinspired optoelectronic memristor incorporating two antagonistic photovoltaic junctions can autonomously adjust its response to varying light stimuli, enabling the amplification of shadows and inhibition of highlight saturation. The memristor arrays are capable of capturing a high‐contrast scene up to 94 dB with a faster active adaptation speed (less than 1.2 s) than that of biological systems.

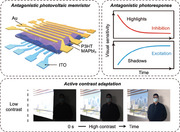

## 1 Introduction

Machine vision systems that emulate biological systems for live monitoring and identification tasks operate under a wide range of illumination conditions.^[^ ^1^, ^2^, ^3^, ^4^, ^5^ ^]^ The high‐fidelity image capture under diverse light illumination is crucial for accurate perception and recognition of the environment, as bright and dim light within a single visual scene can span a large range of 100 dB in intensity.^[^ ^6^, ^7^ ^]^ Due to the predefined internal chemical doping profile and limited well capacity, conventional image sensors based on silicon complementary metal‐oxide semiconductor technology usually have a fixed photoresponsivity and a narrow dynamic range of 60–70 dB.^[^ ^8^, ^9^ ^]^ This sensing constraint severely limits their capability to capture natural scenes that exhibit high contrast and greatly deteriorates the subsequent pattern recognition accuracy using a machine‐learning algorithm.^[^ ^10^ ^]^ To expand the dynamic range, researchers have explored the use of nonlinear compression, multiple exposure sampling, split photodiodes, and saturation detection, but these approaches typically require complex circuitry and algorithms for calibration and post‐processing.^[^ ^11^, ^12^, ^13^ ^]^

Unlike the traditional optical sensors that are limited to fixed responsivity, the human retina can autonomously adapt its sensitivity to varying light stimuli through information preprocessing, permitting the amplification required to detect objects in shadows while avoiding the response saturation to highlights.^[^ ^14^, ^15^, ^16^ ^]^ This active regulation allows to capture of a natural scene with a high contrast of up to 100 dB, although individual neurons in the retina can represent only a limited dynamic range (40 dB) with their range of membrane potentials.^[^ ^6^, ^17^ ^]^ The light‐intensity‐dependent antagonistic photoresponses (excitation and inhibition) that rely on two types of neuronal subsystems (rods and cones) are the key mechanisms involved in active contrast adaptation.^[^ ^18^, ^19^ ^]^ In principle, neuromorphic devices that emulate the biological architectures and functionalities of the human retina may lead to the realization of intelligent visual adaptation photosensors.^[^ ^20^, ^21^ ^]^ Recently, various strategies, including photovoltaic divider,^[^ ^21^ ^]^ halide phase segregation,^[^ ^15^ ^]^ ion migration,^[^ ^10^, ^22^ ^]^ and charge trapping/detrapping,^[^ ^16^, ^23^, ^24^, ^25^ ^]^ have been explored to construct biomimetic devices that can autonomously adapt to the average light intensity, including scotopic and photopic adaptation. However, emulating biological contrast adaptation remains challenging since light‐intensity‐dependent antagonistic functionalities of excitation and inhibition are hard to be realized in a single device.

In this work, we report a bioinspired active contrast adaptation memristor that consists of two antagonistic photovoltaic junctions. Following an increase in light intensity, without the application of an external electrical field, the polarities of short‐circuit current (*I*_sc_) of the optoelectronic memristor can be switched by the photoinduced doping effect at the p‐n junction with asymmetrical profile. The light‐intensity‐dependent switchable photovoltaic characteristics allow two antagonistic functionalities of excitation and inhibition to be achieved in a single device, enabling both amplification of shadows and inhibition of highlights saturation. The photosensitivity of the device follows an inversely proportional relationship to background intensity, matching well with the trend of Weber's law. The memristor arrays are capable of capturing a high‐contrast scene up to 94 dB with a faster active adaptation speed (less than 1.2 s) than that of biological systems. The bio‐inspired self‐powered memristor is highly compatible with high‐density and low‐power consumption machine vision owing to its simple two‐terminal structure and self‐powered paradigm.

## 2 Result and Discussion

### 2.1 Active Contrast Adaptation of the Human Retina

**Figure 1:**
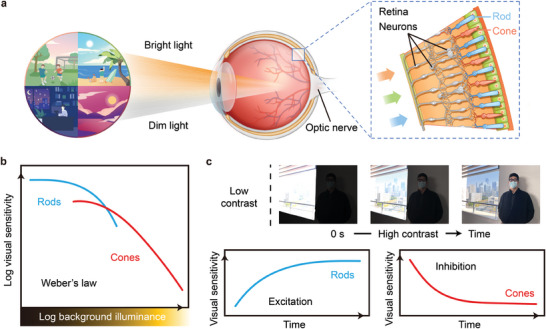
Active contrast adaptation of the human retina. a) Human vision operates under a wide range of illumination conditions in a normal cycle of day and night. The human retina covers this entire range of light intensities with two neuronal subsystems that depend on the activity of two kinds of photoreceptors, rods and cones. b) The relationship of inverse proportionality between sensitivity and light intensity is known as Weber's law. c) Active contrast adaptation of the human retina. Top: schematic of the time course of contrast adaptation by images that include specular highlights and deep shadows. Bottom: the light‐intensity‐dependent antagonistic photoresponses (excitation and inhibition) that rely on two types of neuronal subsystems (rods and cones) are the key mechanism involved in the active contrast adaptation.

Human vision operates under an enormous range of ambient light levels during the normal day‐night cycle. Even specular highlights and deep shadows within a single visual scene can reach a contrast of 100 000:1 (100 dB), which is far beyond the range of output signals retinal neurons can produce (40 dB).^[^ ^6^, ^17^ ^]^ The human retina adapts and perceives such a high‐contrast scene with two neuronal subsystems that depend on the activity of two kinds of photoreceptors, rods and cones (**Figure** **1** **a**).^[^ ^18^, ^19^ ^]^ Rods are highly sensitive to light, and exclusively responsible for vision under dim illumination, whereas cones are much less sensitive and serve daylight vision.^[^ ^26^ ^]^ The mechanism of visual contrast adaptation relies on the ability of photoreceptors themselves to dynamically modulate their sensitivity to different lighting situations.^[^ ^27^, ^28^ ^]^ The photosensitivity (*S*) is inversely proportional to the light intensity (*L*), which is known as Weber's law (Figure 1b).^[^ ^27^, ^28^, ^29^ ^]^ Mathematically, it can be expressed in the following way:

(1) $$\frac{S}{S_{0}}=\frac{L_{0}}{L_{0}+L}\;$$

where *S*_0_ and *L*_0_ are both constants (*S*_0_ represents the sensitivity in darkness, and *L*_0_ is equal to the light intensity required to decrease the sensitivity by one‐half).^[^ ^30^ ^]^ For the light intensity much greater than *L*_0_ (i.e., for *L* ≫ *L*_0_), *S* is approximately equal to (*S*_0_ × *L*_0_)/*L*, an inverse relationship as Weber's law predicts. For rods or cones, Weber's law is strictly followed for the light intensity over at least 2–3 orders of magnitude.^[^ ^29^, ^30^ ^]^ In addition, Weber's law can also be described as a proportional relationship between visual threshold and background intensity (Figure S1, Supporting Information).^[^ ^28^ ^]^ Therefore, as the light intensity becomes weaker (stronger), the retina becomes more (less) sensitive to light stimulus, whereas the visual threshold decreases (increases). Following the switch from a low‐contrast scene to one of high‐contrast, the light‐intensity‐dependent antagonistic functionalities (excitation and inhibition) permit the amplification required to capture the deep shadows while avoiding saturation from the specular highlights (Figure 1c). This active contrast adaptation regulation allows the human retina to encode subtle textures and details in a high‐contrast scene with greater fidelity than a conventional camera at a single exposure sampling.

### 2.2 Antagonistic Photovoltaic Characteristics of the Memristor

**Figure 2:**
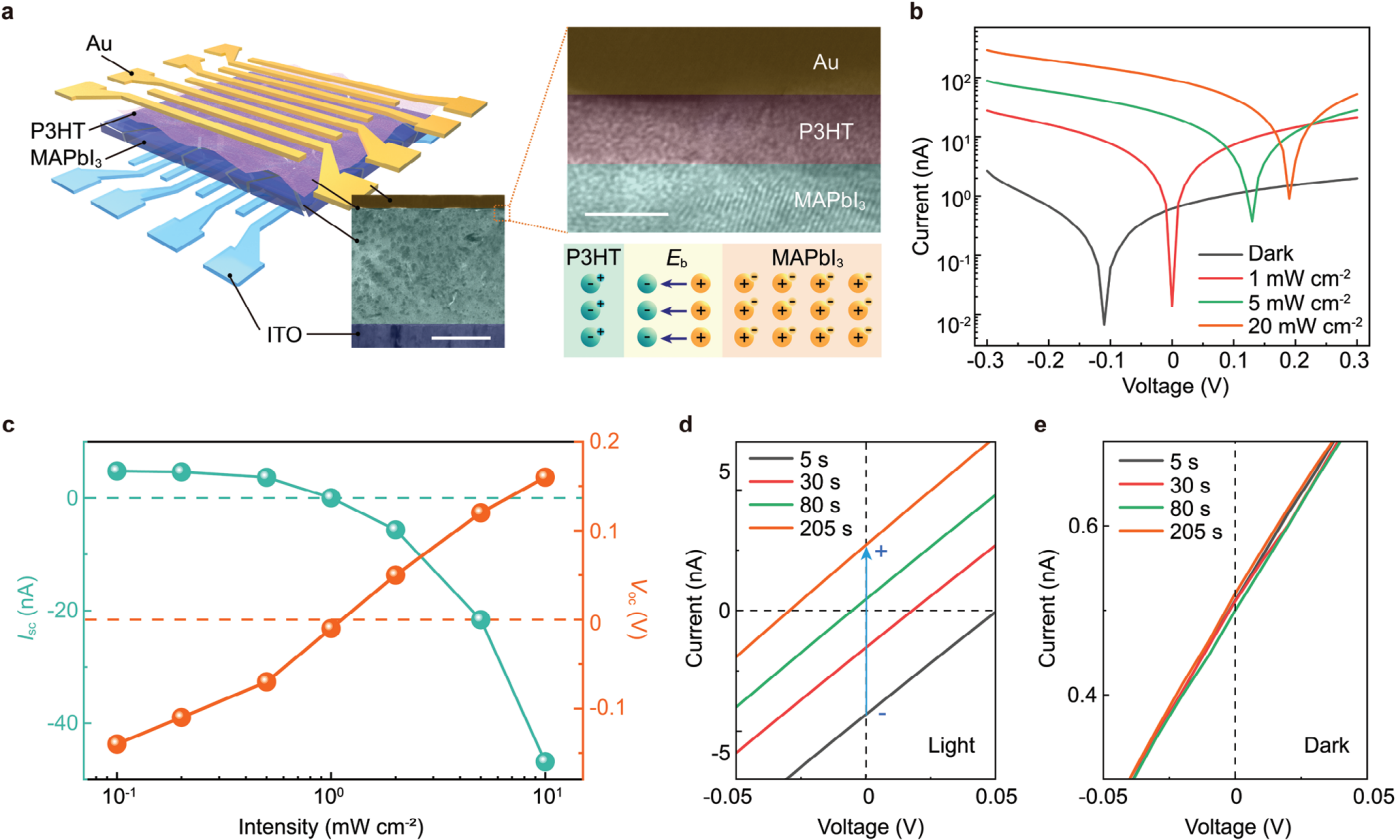
Antagonistic photovoltaic characteristics of the memristor. a) Left: Schematic of ITO/MAPbI_3_/P3HT/Au memristors in an 8 × 8 crossbar array architecture. Middle: False‐coloured cross‐sectional TEM image of the memristor. Scale bar, 250 nm. Top right: Enlarged TEM image at the area marked in the middle panel of a). Scale bar, 5 nm. Bottom right: Schematic of the proposed p‐n junction with the thickness of p‐type P3HT comparable to that of space charge layer. b) Typical *I‐*‐*V* curves of the memristor in the dark and under illumination with varied light intensities (5 s, 626 nm wavelength). c) *I*_sc_ and *V*_oc_ as a function of *L*. d) Typical *I*–*V* curves of the memristor under illumination with different illumination time (1.2 mW cm^−2^, 626 nm wavelength). e) Typical *I‐*‐*V* curves of the memristor in the dark over time.

**Figure** **2** **a** illustrates the self‐powered memristor crossbar array in which individual cross‐point consists of a layered structure of indium tin oxide (ITO)/methylammonium lead iodide (MAPbI_3_)/poly(3‐hexylthiophene‐2,5‐diyl) (P3HT)/Au (left and middle panel of Figure 2a). Cross‐sectional transmission electron microscopy (TEM) images confirm that the thickness of MAPbI_3_ and P3HT films are ≈500 and ≈4 nm, respectively (middle and top right panel of Figure 2a and Figure S2 (Supporting Information). Figure S3 (Supporting Information) presents the atomic force microscopy (AFM) images for analyzing the surface morphology of MAPbI_3_ and MAPbI_3_/P3HT films. UV–vis absorption spectroscopy shows that the absorption range of MAPbI_3_/P3HT films covers the entire UV and visible region with a maximum cut‐off absorption wavelength of ≈780 nm (Figure S4, Supporting Information). The energy‐band alignment of Au, P3HT, MAPbI_3_, and ITO materials is demonstrated in Figure S5 (Supporting Information).^[^ ^31^, ^32^, ^33^ ^]^ It indicates that the self‐powered memristor incorporates two antagonistic photovoltaic junctions (a Schottky junction at ITO/n‐type MAPbI_3_ interface and a p‐n junction at p‐type P3HT/n‐type MAPbI_3_ interface), except for an ohmic contact between p‐type P3HT and Au. Compared with the traditional p‐n junction where the thickness of p‐type and n‐type materials is much larger than that of the space charge region,^[^ ^34^ ^]^ we intentionally control the thickness of p‐type P3HT comparable to that of the space charge layer while keeping the thickness of n‐type MAPbI_3_ much larger than that of the space charge layer (bottom right panel of Figure 2a). This unique asymmetrical interfacial design enables the realization of the photoinduced doping effect at the p‐n junction and offers the possibility of self‐modulated active contrast adaptation in the antagonistic photovoltaic memristor.

Figure 2b illustrates the *I*–*V* characteristics of the optoelectronic memristor in the dark and under illumination with varied light intensities, ranging from 1 to 20 mW cm^−2^ (5 s, 626 nm wavelength). Initially, the device had an *I*_sc_ of 0.61 nA and an open‐circuit voltage (*V*_oc_) of −0.11 V in the dark (black line, Figure 2b). Following an increase in *L* to 1, 5, and 20 mW cm^−2^, *I*_sc_ shifts toward a more negative direction (stepping down to 13.9 pA, −21.6 nA, and −92.0 nA), while *V*_oc_ changes toward a more positive direction (increasing to 0, 0.16, and 0.19 V, respectively). The corresponding linear plots of the *I‐*‐*V* curves in the dark and under different light intensities are shown in Figure S6 (Supporting Information). We extract *I*_sc_ and *V*_oc_ as a function of *L* in Figure 2c. The results indicate that the photovoltaic direction of the memristor is flipped as the light intensity increases. In addition, the device also exhibits illumination‐time (*t*)‐dependent switchable photovoltaic characteristics (Figure 2d). When applying illumination for 5 s with a fixed light intensity (1.2 mW cm^−2^), the device shows an *I*_sc_ of −4.31 nA and a *V*_oc_ of 0.05 V (black line, Figure 2d). However, as the exposure time increases to 205 s, the memristor ultimately shows opposite photovoltaic behavior with an *I*_sc_ of 2.71 nA and a *V*_oc_ of −0.03 V (orange line, Figure 2d). The exposure‐time‐dependent *I*‐*V* characteristics under different light intensities are illustrated in Figure S7 (Supporting Information). From the dependence of *I*_sc_ and *V*_oc_ on *t* (Figure S8, Supporting Information), we observe that *I*_sc_ shifts toward a more positive direction with an increase in *t*, while *V*_oc_ changes toward a more negative direction. By contrast, no obvious *I*–*V* behavior change is observed over time for the memristor in the dark condition (Figure 2e).

### 2.3 Dynamic Photocurrent Characteristics of the Memristor

**Figure 3:**
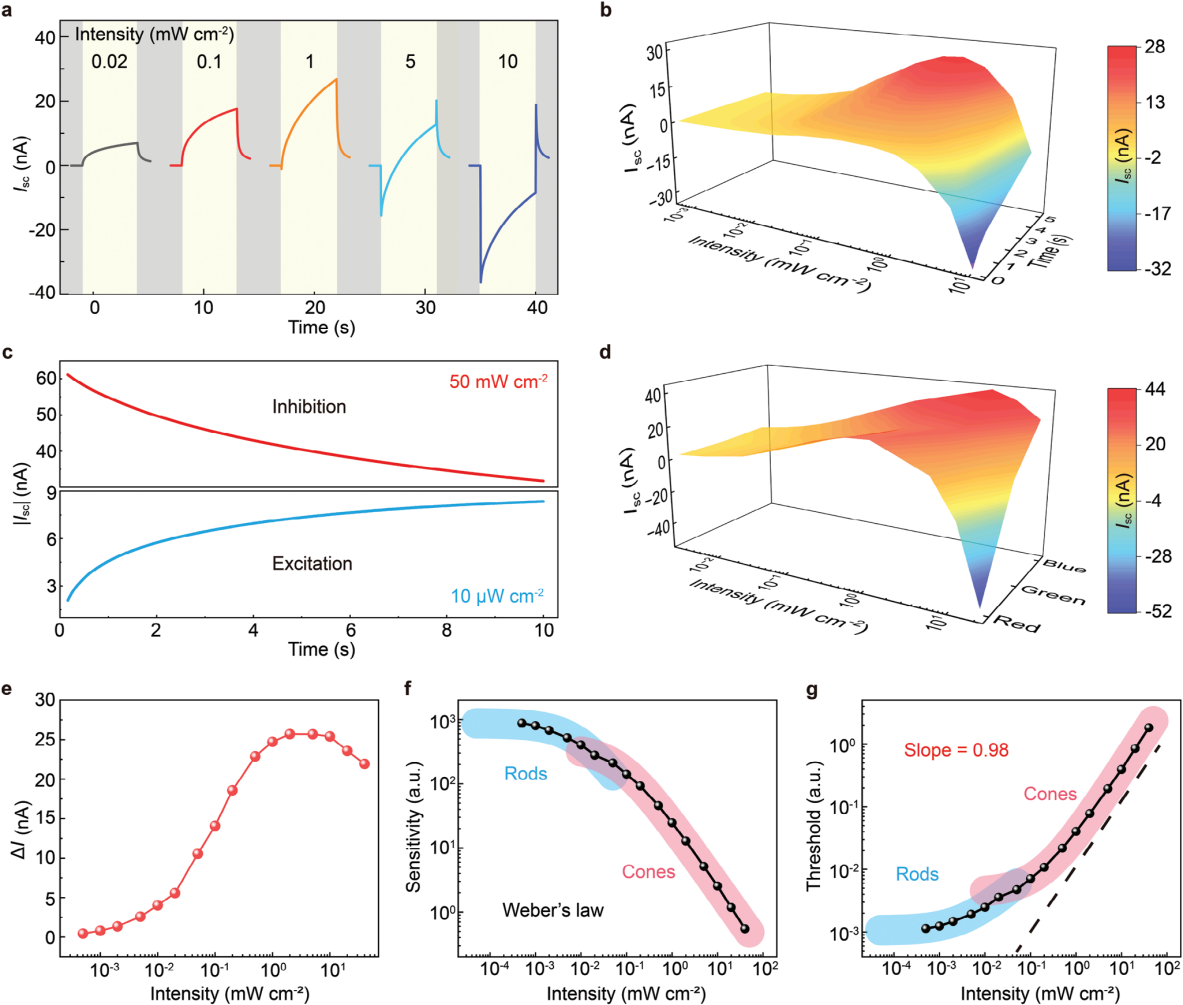
Dynamic photocurrent characteristics of the memristor. a) Real‐time photoresponse (*I*_sc_) of the memristor under illumination with varied light intensities (5 s, 626 nm wavelength). b) Light‐intensity and exposure‐time‐dependent *I*_sc_. c) *I*_sc_ response of the device under continuous illumination of 50 mW cm^−2^ (Top) and 10 µW cm^−2^ (Bottom). d) Light‐intensity and wavelength‐dependent *I*_sc_. Red, 626 nm; green, 519 nm and blue, 458 nm. e) Relationship between Δ*I* and light intensity. f) Relationship between photosensitivity (*S*) and light intensity. g) Visual threshold as a function of background intensity.

We further investigated the dynamic photocurrent characteristics of the antagonistic photovoltaic memristor under short‐circuit conditions without applying any read voltage (i.e., *V*_r_ = 0 V). **Figures** **3** **a** and S9 (Supporting Information) present *I*_sc_ versus illumination time curves under different light intensities (ranging from 0.5 µW cm^−2^ to 40 mW cm^−2^). The memristor exhibits both light‐intensity and illumination‐time‐dependent dynamic photocurrent characteristics (Figure 3b). When the light intensity is below 1 mW cm^−2^, the polarity of *I*_sc_ is positive, and the amplitude of *I*_sc_ increases with an increase in light intensity. For example, the peak photocurrent at 5 s of light exposure (*I*_5_) increases from 0.46 nA at 0.5 µW cm^−2^ to 26.82 nA at 1 mW cm^−2^. The *I*_5_ reaches its maximum value at 1 mW cm^−2^ and subsequently declines as the light intensity continues to increase. The reduction of photocurrent can be attributed to the reversal of photovoltaic direction. When the light intensity is above 1 mW cm^−2^ (including 1 mW cm^−2^), the polarity of the initial transient photocurrent (*I*_i_) switches from positive to negative, and *I*_sc_ shifts toward a more negative direction with a further increase in light intensity. Furthermore, we observe that *I*_sc_ changes toward a more positive direction as the exposure time prolongs, regardless of the light intensity. The switchable photovoltaic characteristics of the memristor enable two antagonistic functionalities of excitation and inhibition to be achieved in a single device. Figure 3c shows the exposure‐time‐dependent *I*_sc_ under two different light intensities. For a bright light intensity of 50 mW cm^−2^, the absolute value of *I*_sc_ gradually decreases over time, which exhibits a current inhibition characteristic (top panel of Figure 3c). In contrast, for a dim light intensity of 10 µW cm^−2^, the absolute value of *I*_sc_ gradually increases over time, which features a current excitation behavior (bottom panel of Figure 3c).

In addition, we studied the effect of P3HT thickness on the dynamic photocurrent characteristics of the memristor. For the device without a P3HT layer (i.e., ITO/MAPbI_3_/Au), the memristor exhibits a negative stable *I*_sc_ over time under illumination with different light intensities, whereas the absolute value of *I*_sc_ increases with an increase in light intensity at a slope of 0.9 on a log‐log scale (Figure S10, Supporting Information). It is worth noting that the light‐intensity‐dependent photovoltaic direction reversal can only be observed at ITO/MAPbI_3_/P3HT/Au structured memristors with P3HT thickness between 2 and 6.5 nm (Figure S11, Supporting Information). Compared with a thinner P3HT thickness (2 nm), a thicker one (4 nm) results in substantially increased *I*_sc_ and requires a larger threshold light intensity (*L*_th_) for reversing the sign of *I*_sc_. Specifically, with the increase in P3HT thickness, the maximum photocurrent (*I*_max_) increases from 1.08 to 26.82 nA, and *L*_th_ increases from 5 to 1 mW cm^−2^. Once the thickness of P3HT is greater than 6.5 nm (including 6.5 nm), the sign of *I*_sc_ is always positive under different light intensities, and the amplitude of *I*_sc_ increases with an increase in light intensity (Figure S12, Supporting Information). Figure 3d and Figure S13 (Supporting Information) compare the dynamic photocurrent characteristics of the memristor under illumination with varied wavelengths. Detailed emission spectra of the used LEDs are provided in Figure S14 (Supporting Information). The peak emission wavelengths of red, green, and blue LEDs are 626, 519, and 458 nm, respectively. The results show that the polarity of *I*_sc_ can be reversed under different wavelengths of illumination. Compared with a longer wavelength (red light, 626 nm), a shorter one (blue light, 458 nm) results in substantially increased *I*_sc_ and requires a larger *L*_th_ for reversing the sign of *I*_sc_. Specifically, as the wavelength becomes shorter, *I*_max_ increases from 8.86 to 43.80 nA, and *L*_th_ increases from 200 to 20 mW cm^−2^. The responsivity (*R*_λ_), spectral detectivity (D*), and noise equivalent power (NEP) of memristors under illumination with varied wavelengths (red light, 626 nm; green light, 519 nm, and blue light, 458 nm) are listed in Table S1 (Supporting Information).

We extract *I*_i_ and *I*_5_ as a function of *L* in Figure S15 (Supporting Information). The photocurrent change (Δ*I*) under continuous illumination of fixed light intensity is defined as Δ*I* = *I*_5_ − *I*_i_. From the dependence of Δ*I* on *L* (Figure 3e), we observe that Δ*I* increases from 0.44 nA at 0.5 µW cm^−2^ to 24.72 nA at 1 mW cm^−2^. The Δ*I* reaches a stable level in the light intensity range of 1 to 10 mW cm^−2^ and subsequently declines slightly with further increase in light intensity. Figure S16 (Supporting Information) shows the relationship between Δ*I* and *L* on a log‐log scale, where the linear dynamic range (LDR) of the memristor obtained by linear fitting is 52 dB. Inspired by the adaptive kinetics of biological photoreceptors,^[^ ^29^, ^30^ ^]^ the photosensitivity is defined as follows:

(2) $$s=\frac{\Delta I}{L}=\frac{I_{5}-I_{i}}{L}\;$$

We extract the relationship between *S* and *L* in Figure 3f. The curve can be well fitted by Weber's law equation: *S*/*S*_0_ = *L*_0_/(*L*_0_ + *L*). The fitted result has two branches connected by a distinct kink: In the light intensity range of 20 µW cm^−2^–40 mW cm^−2^, *S*_0_ = 366.9, and *L*_0_ = 6.6 × 10^−2^, which corresponds to the regime of cone vision; In the light intensity range of 0.5–20 µW cm^−2^, *S*_0_ = 878.8, and *L*_0_ = 9.2 × 10^−3^, which corresponds to the regime of rod vision. By calculating the visual threshold Δ*L* (Δ*L* = 1/*S*), we observe that Δ*L* increases with an increase in *L* at a slope of 0.98 (*k* = 0.98) on a log‐log scale (Figure 3g). These characteristics indicate that the antagonistic photovoltaic memristor fits well with the trend of Weber's law.

### 2.4 Working Mechanism of the Memristor

**Figure 4:**
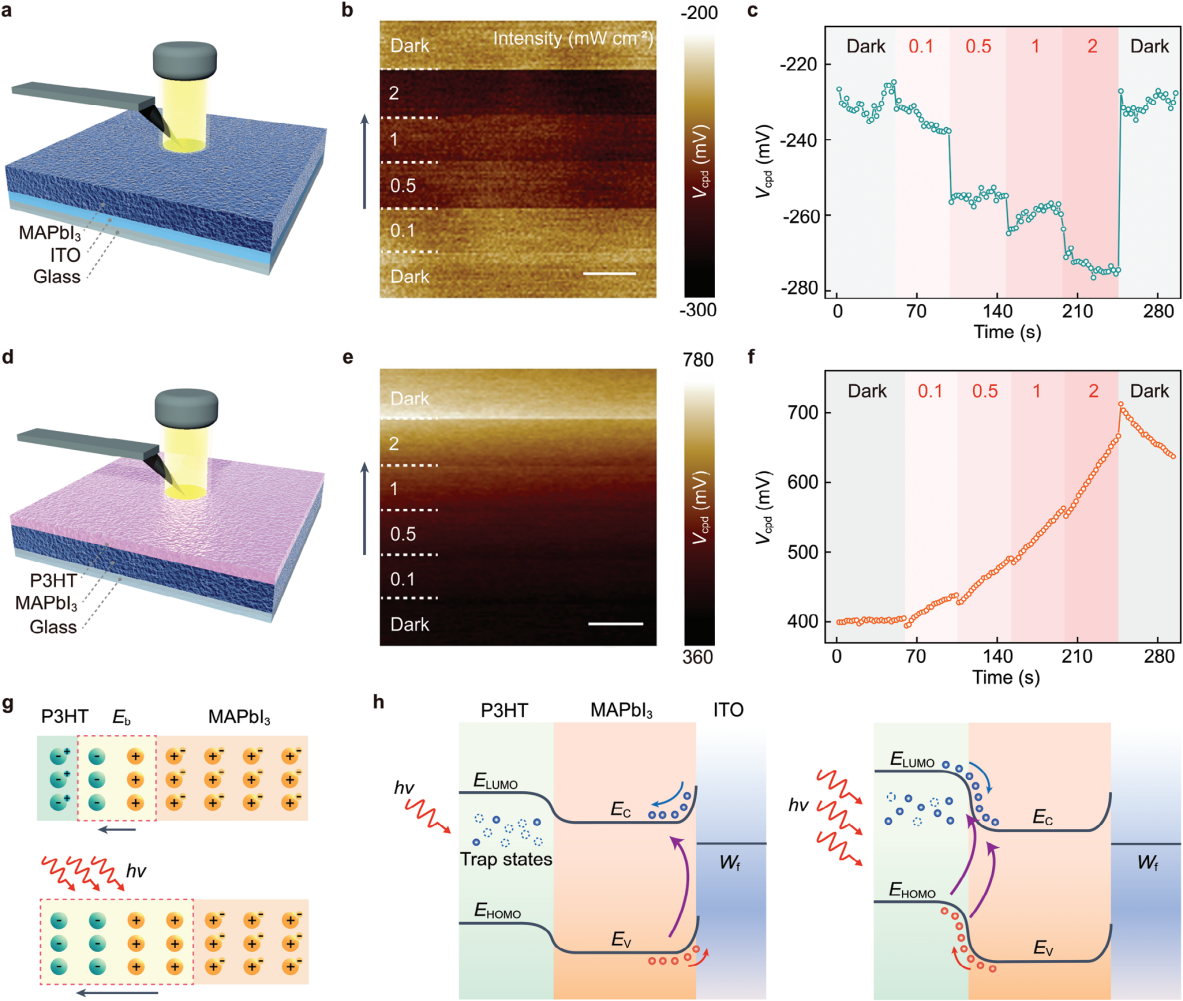
Working mechanism of the memristor. a) Schematic of KPFM characterization of ITO/MAPbI_3_ device. b) Surface potential profile of ITO/MAPbI_3_ device illuminated with a series of light intensity steps. The arrow indicates the time sequence of the test. Scale bar, 500 nm. c) Real‐time *V*_cpd_ change extracted from b). d) Schematic of KPFM characterization of MAPbI_3_/P3HT device. e) Surface potential profile of MAPbI_3_/P3HT device illuminated with a series of light intensity steps. Scale bar, 500 nm. f) Real‐time *V*_cpd_ change extracted from e). g) Schematic of photoinduced charge tunneling at the asymmetrical p‐n junction under illumination, leading to n‐doping on MAPbI_3_ side and p‐doping on P3HT side. h) Energy‐band alignment of the device under low light intensity (left) and high light intensity (right), showing different charge transport direction.

The ITO/MAPbI_3_/Au device shows a negative stable *I*_sc_ upon fixed light illumination, which suggests that the Schottky junction at the Au/n‐type MAPbI_3_ interface dominates the photovoltaic direction (Figure S17, Supporting Information).^[^ ^35^ ^]^ The introduction of a P3HT layer between Au and MAPbI_3_ turns the Schottky junction into a p‐n junction (P3HT/MAPbI_3_) and an ohmic contact (P3HT/Au). Given that a similar dynamic photocurrent behavior is observed at ITO/MAPbI_3_/P3HT/ITO device by replacing Au with ITO electrodes (Figure S18, Supporting Information), we can conclude that the switchable photovoltaic characteristics of the memristor are determined by the antagonism of Schottky junction (ITO/MAPbI_3_) and p‐n junction (P3HT/MAPbI_3_). To identify the specific roles of two photovoltaic junctions, we conducted Kelvin probe force microscopy (KPFM) measurements (**Figure** **4** **a,d**). When the ITO/MAPbI_3_ device is illuminated with a series of light intensity steps (red light, 626 nm), the local surface potential (*V*_cpd_) first declines sharply with each increase in light intensity, and then remains unchanged within the subsequent illumination time (Figure 4b,c). The drop of *V*_cpd_ under illumination can be attributed to the built‐in electric field of the ITO/MAPbI_3_ Schottky junction, which makes photogenerated electrons spontaneously separate from the MAPbI_3_ side. After removing the light irradiation, the *V*_cpd_ rapidly returns to its initial value.

Interestingly, for the MAPbI_3_/P3HT device, an antagonistic *V*_cpd_ change is observed as the light intensity increases in steps (Figure 4e,f). With each increase in light intensity, the *V*_cpd_ first drops sharply at the beginning of light irradiation and then increases gradually within the subsequent illumination time. In contrast, after turning off the light irradiation, the *V*_cpd_ first increases sharply and then decreases gradually to its initial value after 20 min (Figure S19, Supporting Information). Increasing the light intensity from 0.1 to 1 mW cm^−2^ results in a significant decrease of transient *V*_cpd_ at the beginning of light irradiation with Δ*V*_cpd_ changing from 8 to 30 mV (Figure S20, Supporting Information). The sharp drop of *V*_cpd_ suggests that the negatively charged electrons in MAPbI_3_ can tunnel to the P3HT side under illumination, which leaves the positively charged ions to n‐dope MAPbI_3_. Similarly, the positively charged holes in P3HT can tunnel to the MAPbI_3_ side, leaving the negatively charged ions to p‐dope P3HT. This photoinduced doping can modulate the interface barrier and potential profile,^[^ ^36^, ^37^, ^38^ ^]^ leading to an increased built‐in electric field at the asymmetrical p‐n junction under illumination (Figure 4g). On the other hand, the gradual change of *V*_cpd_ suggests the existence of trap states in P3HT (Figure S21, Supporting Information).^[^ ^39^, ^40^, ^41^ ^]^ Under light irradiation, these localized states can trap photogenerated electrons, which results in the accumulation of holes and the consequent gradual increase of *V*_cpd_ and *I*_sc_. Figure 4h illustrates the energy‐band alignment of two antagonistic photovoltaic junctions under various illumination conditions, showing different charge transport directions. Under low light intensity, the ITO/MAPbI_3_ Schottky junction dominates the photovoltaic direction. The ITO/MAPbI_3_/P3HT/Au device shows a positive *I*_sc_. Under high light intensity, the p‐n junction dominates the photovoltaic direction. The increased built‐in electric field at the asymmetrical p‐n junction, induced by the photodoping effect, switches the photovoltaic direction from positive to negative.

### 2.5 Mimicking Active Contrast Adaptation Functionality

**Figure 5:**
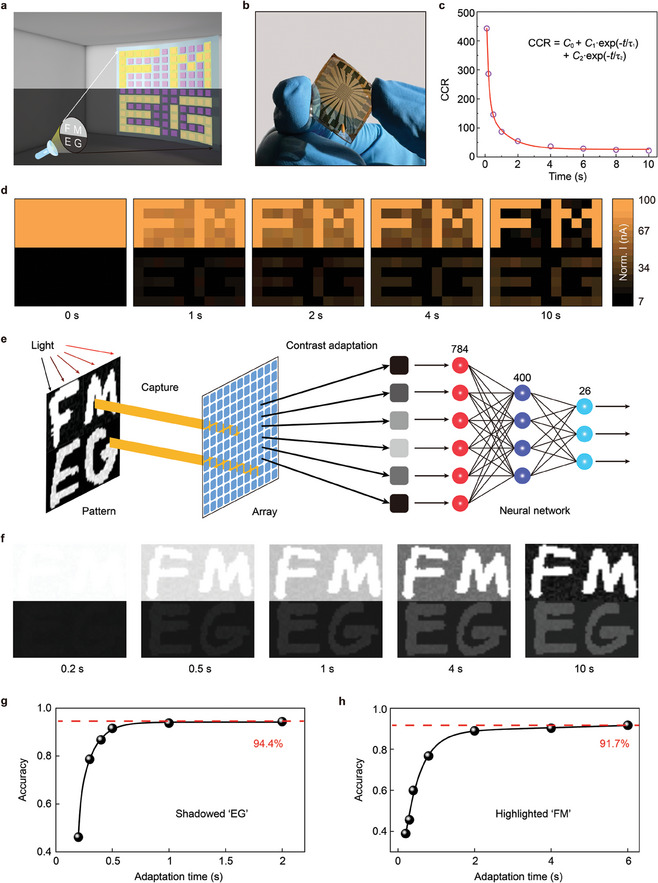
Mimicking active contrast adaptation functionality with memristor arrays. a) Schematic of an 11 × 11 memristor array to encode a high‐contrast “FMEG” pattern (94 dB). b) Photograph of a flexible 11 × 11 pixel array integrated on a 2 × 2 cm^2^ PET substrate, where the area of each pixel is ≈0.01 mm^2^. c) Dynamic CCR change enabled by the antagonistic photoresponse behaviors, where CCR results can be well fitted by a double exponential decay function (red line). d) Time course of active contrast adaptation for the high‐contrast “FMEG” image. e) Illustration of a machine vision system based on a memristor array for active contrast adaptation and an ANN for pattern recognition. f) Temporal evolution of the high‐contrast EMNIST image during contrast adaptation. g) Recognition accuracy of the visual system for the shadowed “EG” pattern with bioinspired vision sensors. h) Recognition accuracy of the visual system for the highlighted “FM” pattern with bioinspired vision sensors.

In the biological visual system, individual retinal neurons can produce only a limited range of output signals.^[^ ^6^, ^17^ ^]^ To encode a high‐contrast natural scene, the human retina employs an active contrast adaptation strategy, which allows shadows to be amplified while inhibiting highlights saturation.^[^ ^6^, ^14^ ^]^ Based on the light‐intensity‐dependent antagonistic photoresponse characteristics of the optoelectronic memristor, we can mimic this active contrast adaptation functionality with memristor arrays. A flexible 11 × 11 device array was fabricated by integrating the memristor crossbar on a 2 × 2 cm^2^ PET substrate (**Figure** **5**a,b). Figure S22 (Supporting Information) presents the photocurrent properties of all memristor pixels, showing that 103 of the 121 devices (85.12%) exhibit uniform *I*_sc_ with relatively small variation. The image sensing and adaptation function was examined by projecting a high‐contrast pattern of “FMEG” onto the memristor array. As shown in Figure 5a, the character “EG” (10 µW cm^−2^) in the lower half is under a dim‐light background (1 µW cm^−2^), and the character “FM” (50 mW cm^−2^) in the upper half is under a bright‐light background (25 mW cm^−2^). The optical pattern that consists of shadowed “EG” and highlighted “FM” offers a contrast as high as 50 000:1 (94 dB).

Figure 5c shows the dynamic current compression effect enabled by the antagonistic photoresponse behaviors. Here, we define the current compression ratio (CCR) of the memristor array as follows:

(3) $$\mathrm{CCR}\;=\frac{I_{\max}^{*}}{I_{\min}^{*}}\;$$

where $I_{\max}^{*}$ is the average *I*_sc_ value under the maximum light intensity of 50 mW cm^−2^, and $I_{\min}^{*}$ is the average *I*_sc_ value under the minimum light intensity of 1 µW cm^−2^ (Figure S23, Supporting Information). The CCR results can be well fitted by a double exponential decay function: CCR = *C*_0_ + *C*_1_ × exp(−*t*/*τ*_1_) + *C*_2_ × exp(−*t*/*τ*_2_). The obtained adaptation speed, including a fast adaptation constant *τ*_1_ = 0.13 s and a slow adaptation constant *τ*_2_ = 1.14 s, is comparable to that in the biological retina.^[^ ^19^ ^]^ Figure 5d shows the evolution of the reconstructed high‐contrast “FMEG” image over time (Figure S24 (Supporting Information) depicts the specific test methods and results). Interestingly, with the active contrast adaptation, the initially featureless image gradually becomes a spatially resolved “FMEG” pattern. To quantitatively evaluate the image contrast, the photocurrent value was converted to a grey level between 0 and 255 (Figure S25, Supporting Information). For the shadowed “EG” pattern, the image contrast increases from 4 at 0 s to 30 (1 s), 56 (4 s), and 71 (10 s). For the highlighted “FM” pattern, the image contrast increases from 0 at 0 s to 64 (1 s), 154 (4 s), and 244 (10 s). The active contrast adaptation regulation enhances the image contrast for both shadowed “EG” and highlighted “FM” patterns.

The high‐fidelity image capture is critical for accurate pattern recognition in machine vision applications. To quantitatively evaluate the effect of contrast adaptation on improving pattern recognition, a machine vision system composed of a memristor array and an artificial neural network (ANN) is constructed (Figure 5e). The vision system is first trained and tested by the Extended Modified National Institute of Standards and Technology (EMNIST) dataset in a normal environment to verify the recognition capability of ANN. Then, the treated high‐contrast image is projected onto the memristor array for perception and adaptation. After that, the modified image is fed into ANN for pattern recognition. Detailed simulation process and ANN architecture are described in Table S2 (Supporting Information) and Experimental Section. Figure 5f illustrates the temporal evolution of the high‐contrast EMNIST image during contrast adaptation, showing obvious improvements in the image contrast as the time increases from 0.2 to 10 s. Figure 5g,h respectively show the recognition accuracy of the visual systems for the shadowed “EG” and highlighted “FM” patterns. With contrast adaptation, the recognition accuracy improves from 46.1% at 0.2 s to 94.4% at 2 s for shadowed “EG”, and improves from 38.9% at 0.2 s to 91.7% at 6 s for highlighted “FM”. In contrast, conventional image sensors with fixed photoresponsivity show a much lower recognition accuracy (17.3% for “EG” and 2.1% for “FM” at 6 s) because of the lack of intrinsic adaptivity to the high‐contrast image (Figure S26, Supporting Information). Besides the demonstration of contrast adaptation functionality, we also examine the active adaptation capability to the average light intensity, including scotopic and photopic adaptation (Figures S27 and S28, Supporting Information). For scotopic adaptation, the recognition accuracy of the visual system increases from 50.4% at 0.2 s to 93.0% at 6 s. For photopic adaptation, the recognition accuracy increases from 23.3% at 0.2 s to 94.0% at 6 s. Detailed performance comparisons of various visual adaptation studies are listed in Table S3 (Supporting Information). The unusual ability of self‐powered operation and exceptional contrast adaptation performance (less than 1.2 s at 94 dB) could promise our device an ultralow energy consumption and excellent adaptivity to the high‐contrast environment.

## 3 Conclusion

We have reported a bioinspired optoelectronic memristor that consists of two antagonistic photovoltaic junctions and can emulate visual scotopic, photopic, and contrast adaptation functionalities. Due to the coupled photodoping and electron trapping effect, the device exhibits light‐intensity‐dependent antagonistic photo‐responses (excitation and inhibition), enabling shadows to be amplified while inhibiting highlights saturation. The photosensitivity of the optoelectronic memristor is inversely proportional to the light stimuli, matching well with the trend of Weber's law. The memristor array can encode high‐contrast scenes up to 94 dB with a simplified structure, higher energy efficiency, and faster adaptation speed (less than 1.2 s). This work can contribute to the development of next‐generation intelligent and low‐power perceptual systems.

## 4 Experimental Section

### Fabrication of the Optoelectronic Memristor Array

First, the ITO bottom electrodes (100 µm in width and 185 nm in thickness) were deposited on the precleaned glass or PET substrates by magnetron sputtering technique, and a shadow mask was utilized to achieve an 11 × 11 array with a total area of 2 × 2 cm^2^. After 30 min of UV‐ozone treatment on the substrates, the MAPbI_3_ precursor solution was spin‐coated onto the ITO electrodes using a one‐step antisolvent method at 4000 r.p.m. for 30 s, during which 300 µL chlorobenzene was dropped to improve the crystalline quality of the perovskite. The MAPbI_3_ precursor solution was prepared according to the method described in the previous work.^[^ ^32^ ^]^ Then, the fabricated perovskite films were annealed at 100 °C for 10 min, resulting in a 500‐nm‐thick active layer. Subsequently, a 4‐nm‐thick P3HT (4000 r.p.m. for 30 s, 2.5 mg mL^−1^ in 1,2‐dichlorobenzene) layer was spin‐coated onto the perovskite layer and annealed at 100 °C for 30 min. Finally, the Au top electrodes (100 µm in width and 50 nm in thickness) were deposited onto the P3HT layer by thermal evaporation. All of the above operations were performed in a glovebox with a nitrogen atmosphere.

### Device Characterization

The cross‐sectional images of the memristor were characterized by TEM (Titan Cubed Themis G2300, FEI). The surface morphology and potential of the devices were analyzed by a scanning probe microscope (Bruker Dimension Icon). The KPFM measurements were performed using a tip coated with Pt‐Ir thin film (SCM‐PIT‐V2) in the SKPM mode at a zero‐bias voltage. The optical absorption property of the thin films was characterized by a UV–vis spectrometer (Agilent Cary 60). All electrical characteristics were measured using an Agilent B2902 system in a glovebox with a nitrogen atmosphere. For the photoresponse measurements, commercially available light‐emitting diodes (LEDs) with red (626 nm), green (519 nm), and blue (458 nm) emissions were used as the light sources. An optical power meter (Newport 1919‐R) was used to calibrate the illumination intensity. The direction of positive photocurrent was defined to be from the Au to the ITO electrode.

### Simulation of ANN

The ANN follows the back‐propagation algorithm. The optoelectronic memristor array size was 28 × 28 and the ANN specification was 784 × 400 × 26. In the simulation, the ANN was first trained and tested by the EMNIST dataset (handwriting letters from “A” to “Z”, including 188958 training patterns and 31346 testing patterns) in a normal environment to verify the recognition capability. Then, the testing pattern was exposed to a high‐contrast or dim (bright) environment. After the contrast adaptation or scotopic (photopic) adaptation in the memristor array, the modified image was fed into ANN for pattern recognition. The obtained recognition accuracy was for the whole EMNIST dataset.

## Conflict of Interest

The authors declare no conflict of interest.

## Supporting information



Supporting Information

## Data Availability

The data that support the findings of this study are available from the corresponding author upon reasonable request.
