# Supplementary material for: An Antagonistic Photovoltaic Memristor for Bioinspired Active Contrast Adaptation
Source: Adv Mater. 2024 Oct 30;36(50):2409844. doi: 10.1002/adma.202409844 (PMC11635913; doi:10.1002/adma.202409844)
Supplement: Supplementary file 1 — Supporting Information [file ADMA-36-2409844-s001.docx]

Supporting Information

An antagonistic photovoltaic memristor for bioinspired active contrast adaptation

*Guodong Gong, You Zhou, Ziyu Xiong, Tao Sun, Huaxin Li, Qingxiu Li, Wenyu Zhao, Guohua Zhang, Yongbiao Zhai, Ziyu Lv, Hongwei Tan, Ye Zhou and Su-Ting Han**

G. Gong, Y. Zhou, T. Sun, H. Li, Q. Li, W. Zhao

Institute of Microscale Optoelectronics

Shenzhen University

Shenzhen 518060, P. R. China

G. Gong, Y. Zhai, Z. Lv

College of Electronics and Information Engineering

Shenzhen University

Shenzhen 518060, P. R. China

Z. Xiong

Institute of Polymer Optoelectronic Materials and Devices

State Key Laboratory of Luminescent Materials and Devices

South China University of Technology

Guangzhou 510640, P. R. China

G. Zhang

Key Laboratory of Physics and Technology for Advanced Batteries (Ministry of Education)

College of Physics

Jilin University

Changchun 130012, P. R. China

H. Tan

NanoSpin

Department of Applied Physics

Aalto University School of Science

P.O. Box 15100, FI-00076 Aalto, Finland

Y. Zhou

Institute for Advanced Study

Shenzhen University

Shenzhen 518060, P. R. China

S.-T. Han

Department of Applied Biology and Chemical Technology

The Hong Kong Polytechnic University

Hung Hom, Kowloon, Hong Kong 999077, P. R. China

E-mail: [suting.han@polyu.edu.hk](mailto:suting.han@polyu.edu.hk)

**
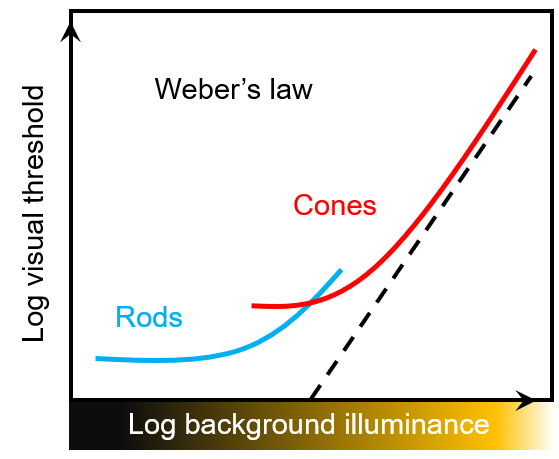
**

**Figure S1.** The visual threshold is proportional to the background intensity, which is known as Weber’s law. The visual threshold is the minimum stimuli intensity required for a person to detect a stimulus. Mathematically, Weber’s law can be described as Δ*P* = *k* × *P*, where Δ*P* is the just perceived change in stimuli, *P* is the background stimulus, and *k* is a constant.

**
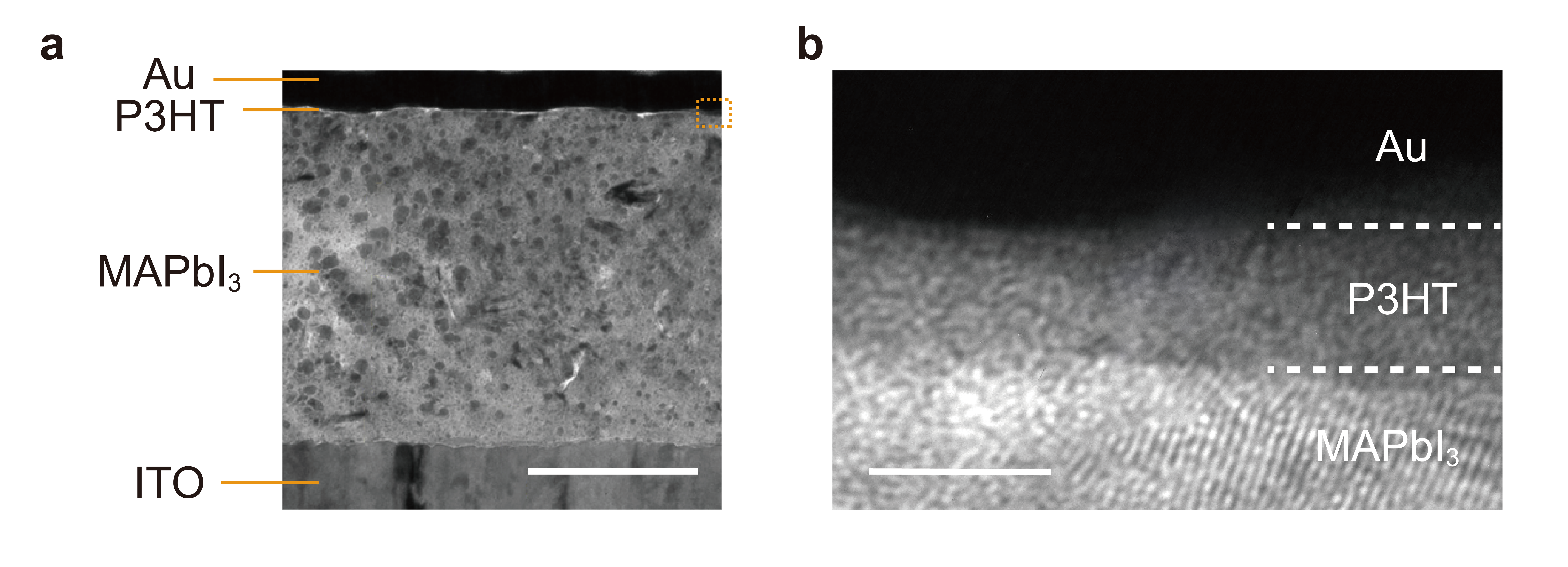
**

**Figure S2.** a) The cross-sectional TEM image of the ITO/MAPbI_3_/P3HT/Au memristor. Scale bar, 250 nm. b) Enlarged TEM image at the area marked in a). Scale bar, 5 nm.

**
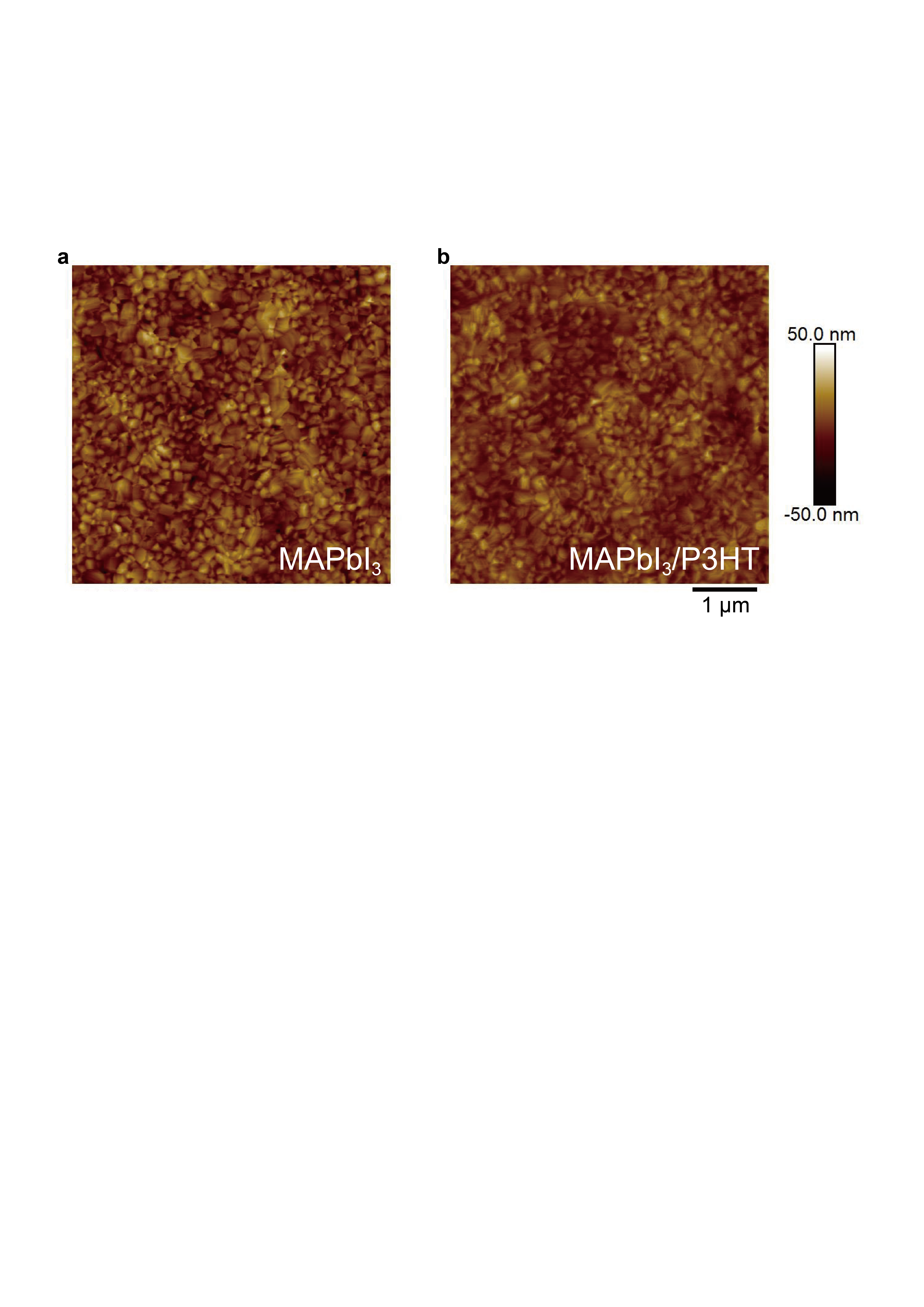
**

**Figure S3.** AFM images of the surface morphology. a) MAPbI_3_ and b) MAPbI_3_/P3HT. The root-mean-square (*R*_q_) roughness of the as-fabricated MAPbI_3_ and MAPbI_3_/P3HT thin films is calculated to be 9.24 and 7.2 nm, respectively.

**
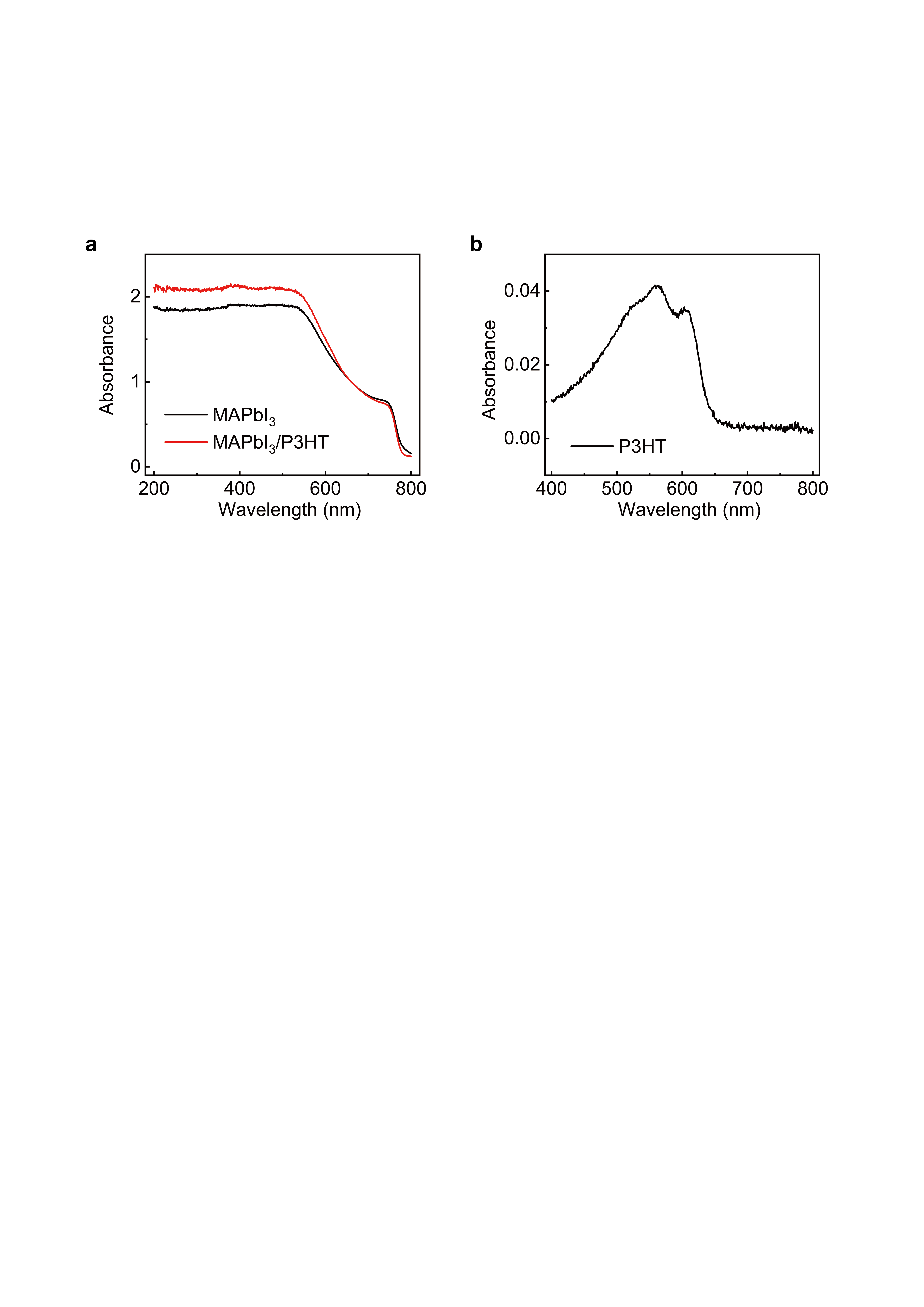
**

**Figure S4.** UV-vis absorption spectra of the thin films. a) MAPbI_3_ and MAPbI_3_/P3HT. b) P3HT. The absorption ranges of MAPbI_3_ and MAPbI_3_/P3HT films both cover the entire UV and visible region with a maximum cut-off absorption wavelength of ~780 nm. The P3HT thin film has a weak absorption band in the visible region.

**
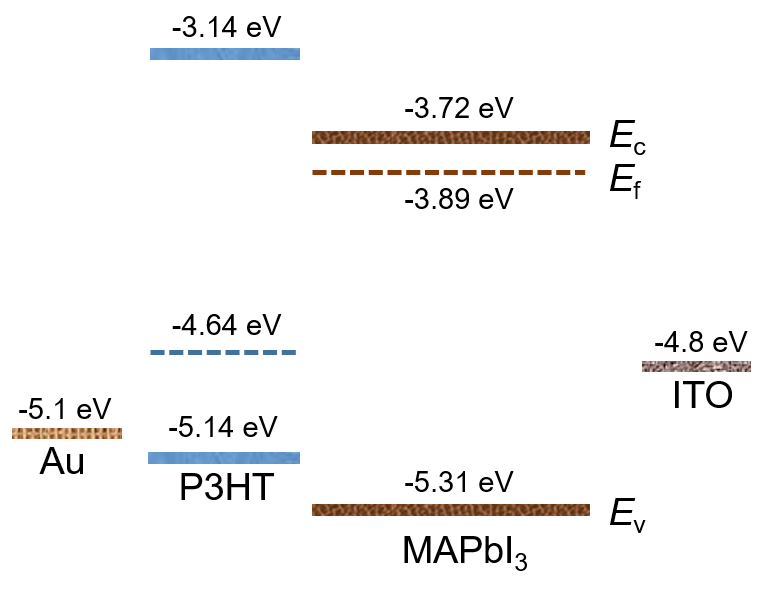
**

**Figure S5.** The energy-band alignment of Au, P3HT, MAPbI_3_, and ITO.

**
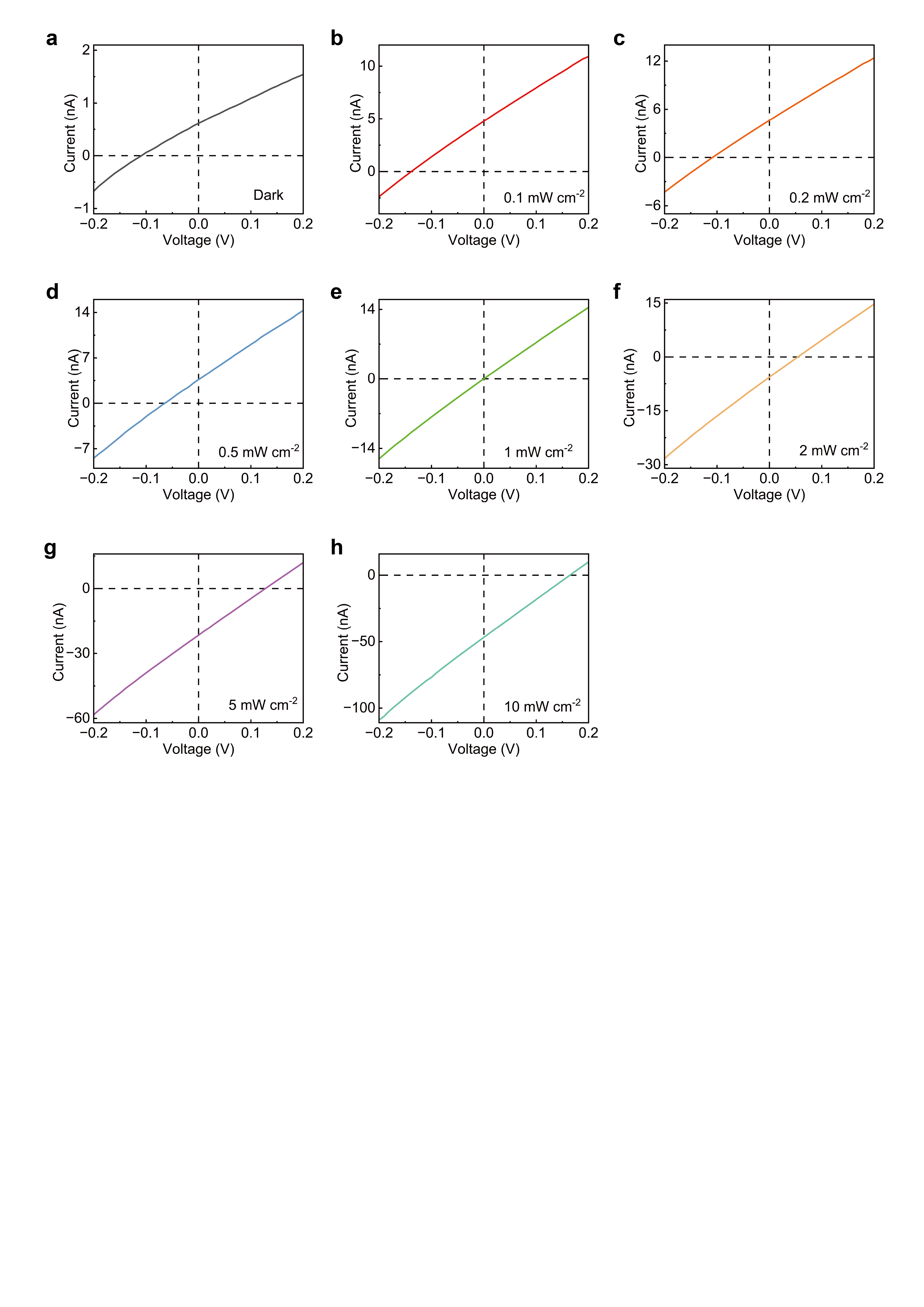
**

**Figure S6.** The linear plot of the *I*-*V* curves in the dark and under illumination with varied light intensities. a) dark. b) 0.1 mW cm^−2^. c) 0.2 mW cm^−2^. d) 0.5 mW cm^−2^. e) 1 mW cm^−2^. f) 2 mW cm^−2^. g) 5 mW cm^−2^. h) 10 mW cm^−2^.

**
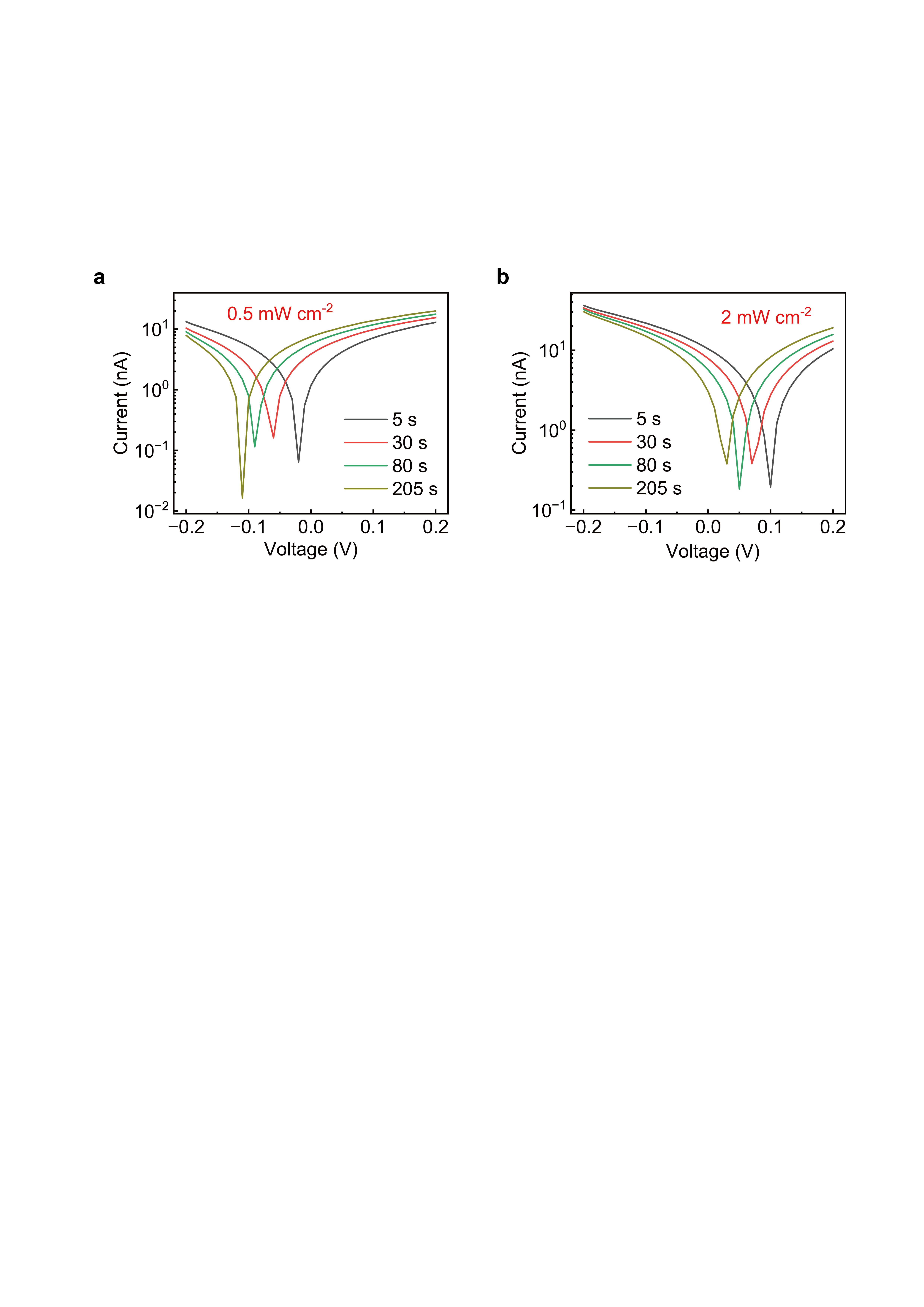
**

**Figure S7.** The exposure-time-dependent *I*-*V* characteristics under different light intensities. a) 0.5 mW cm^−2^ and b) 2 mW cm^−2^.

**
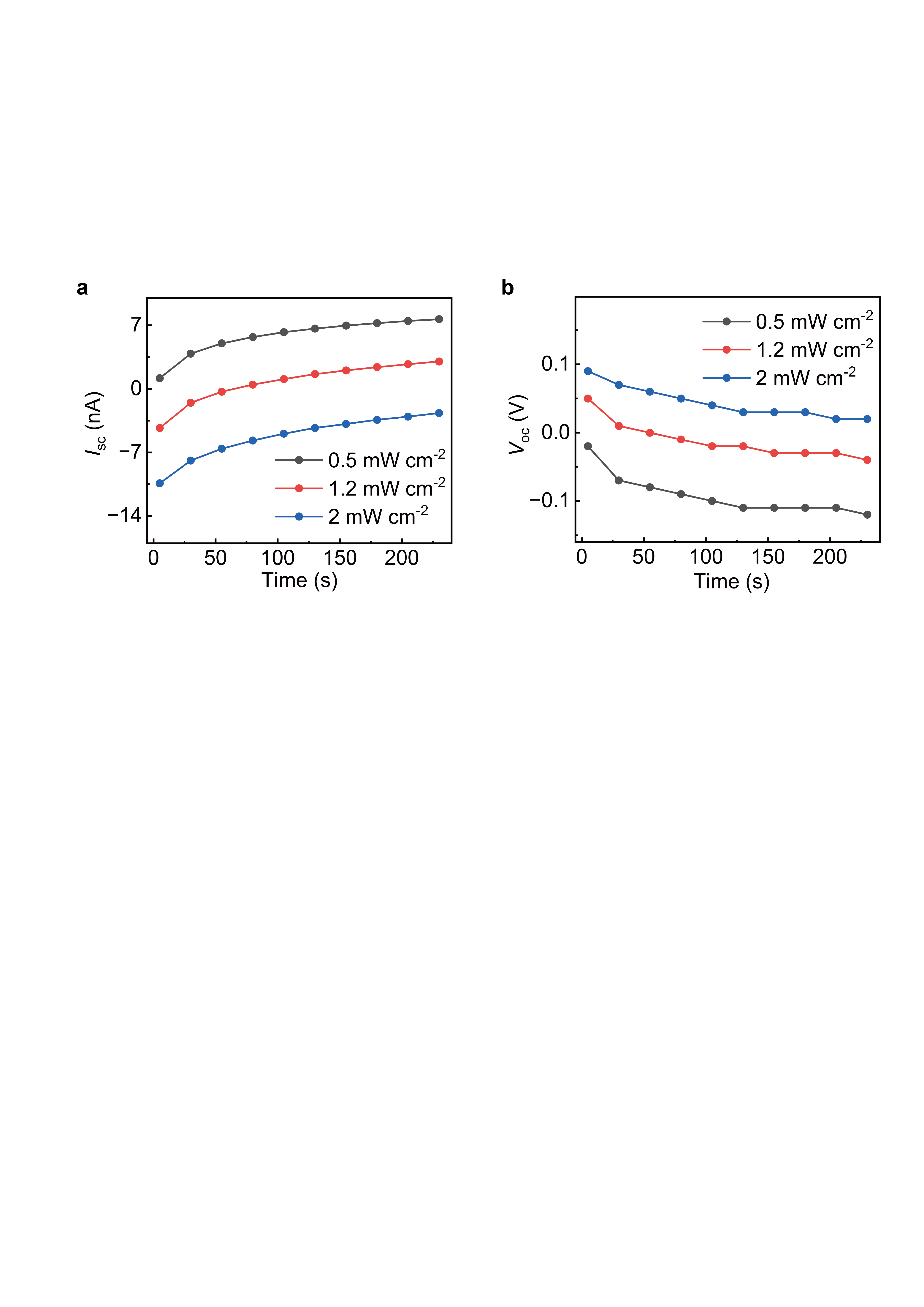
**

**Figure S8.** *I*_sc_ and *V*_oc_ versus exposure time under different light intensities. a) *I*_sc_ and b) *V*_oc_.

**
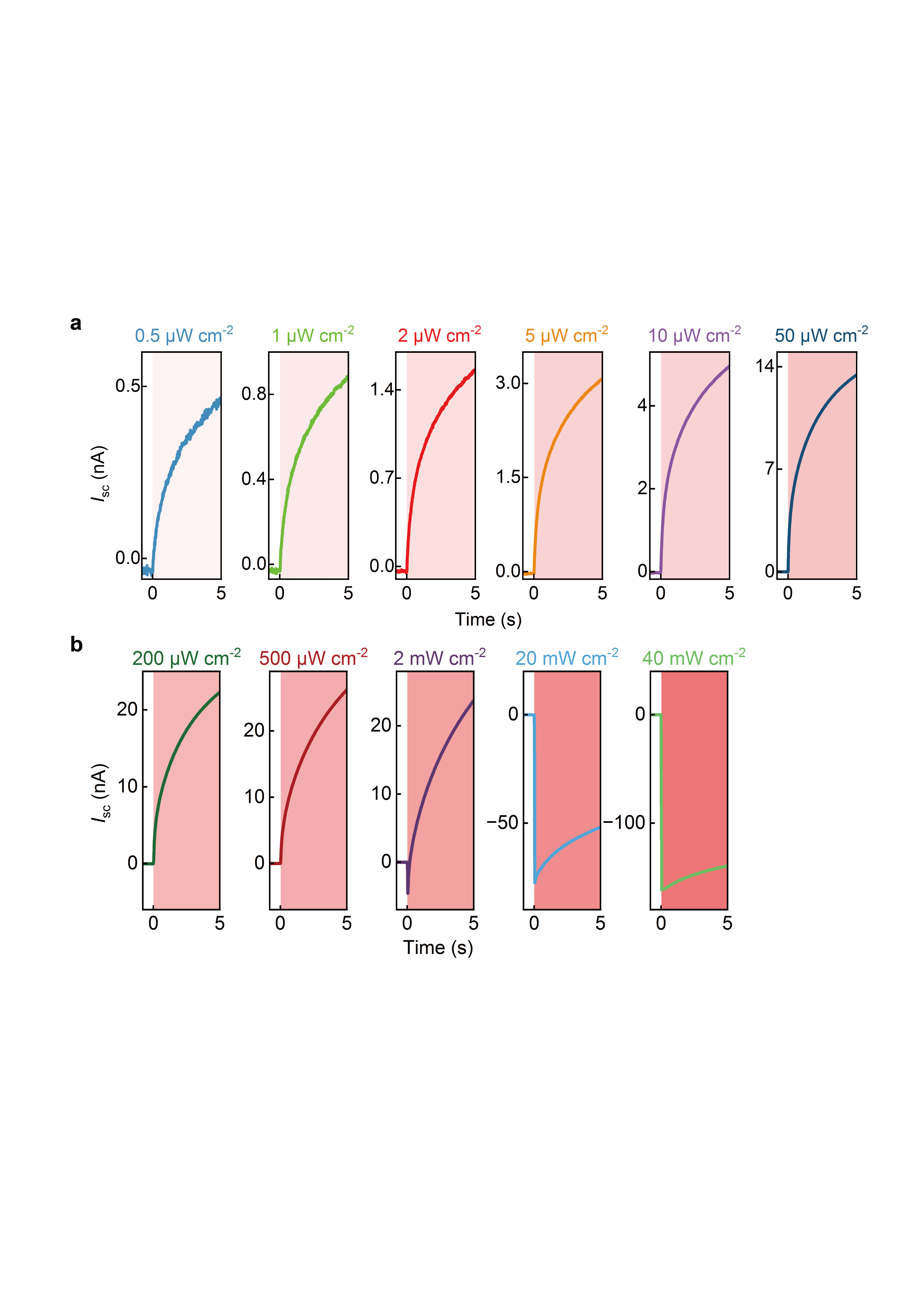
**

**Figure S9.** *I*_sc_ versus illumination time curves under different light intensities (5 s, 626 nm wavelength). a) 0.5 to 50 μW cm^−2^. b) 200 μW cm^−2^ to 40 mW cm^−2^.

**
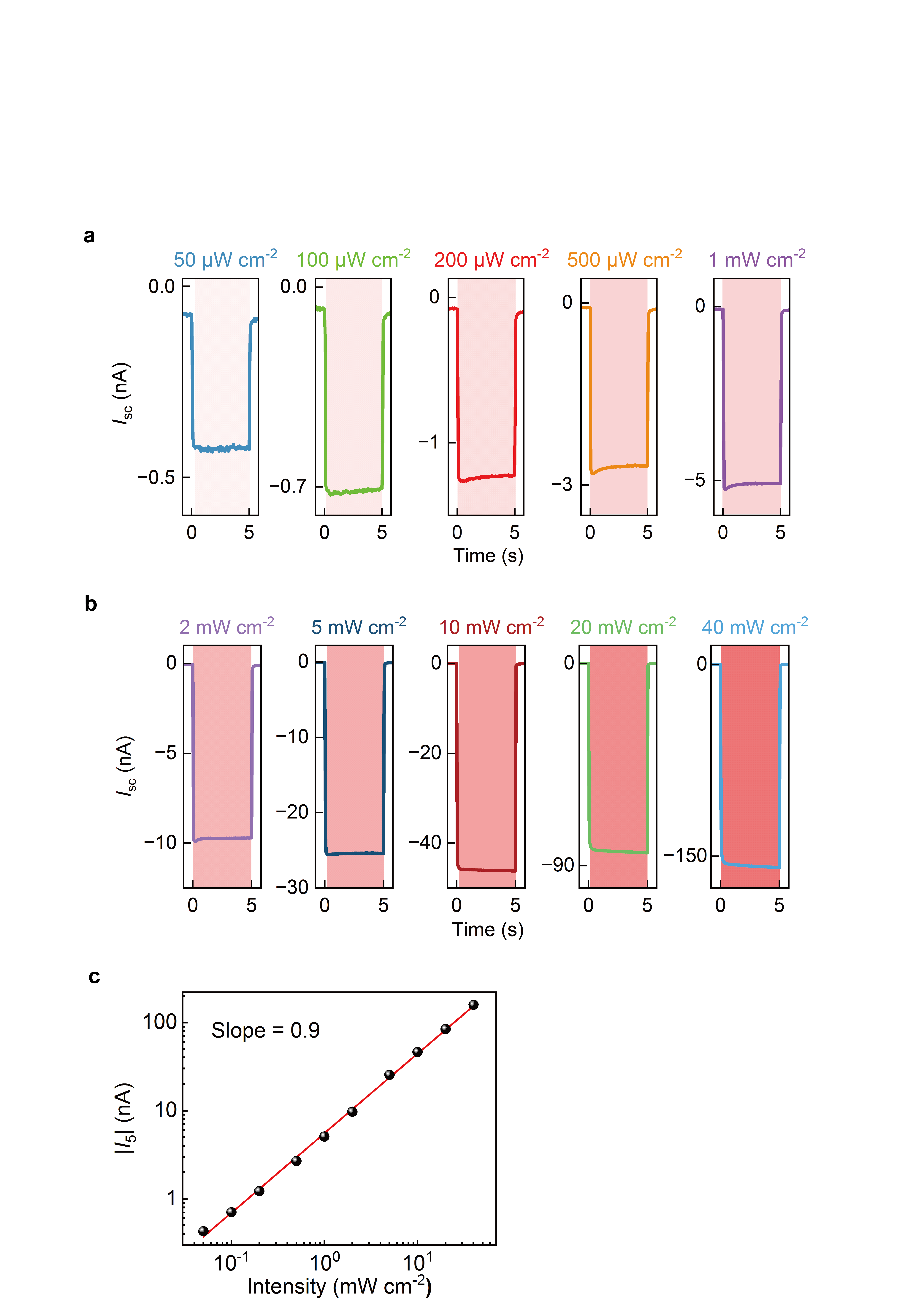
**

**Figure S10.** Dynamic photocurrent characteristics of ITO/MAPbI_3_/Au device under illumination with varied light intensities (5 s, 626 nm wavelength). a) 50 μW cm^−2^ to 1 mW cm^−2^. b) 2 to 40 mW cm^−2^. c) Relationship between absolute value of *I*_5_ and light intensity.

**
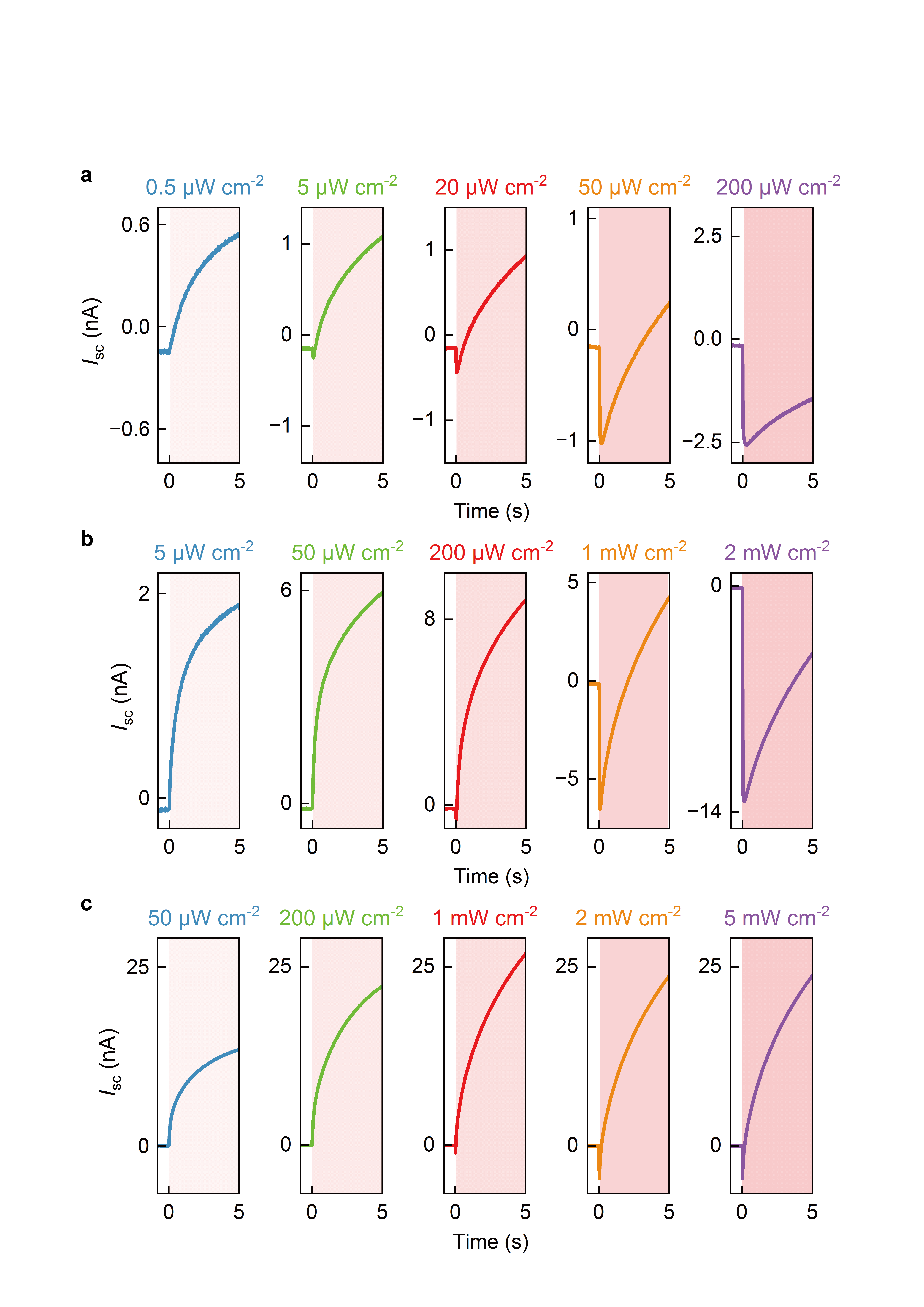
**

**Figure S11.** Effect of P3HT thickness on the dynamic photocurrent characteristics of ITO/MAPbI_3_/P3HT/Au memristors. a) 2 nm. b) 3 nm. c) 4 nm.

**
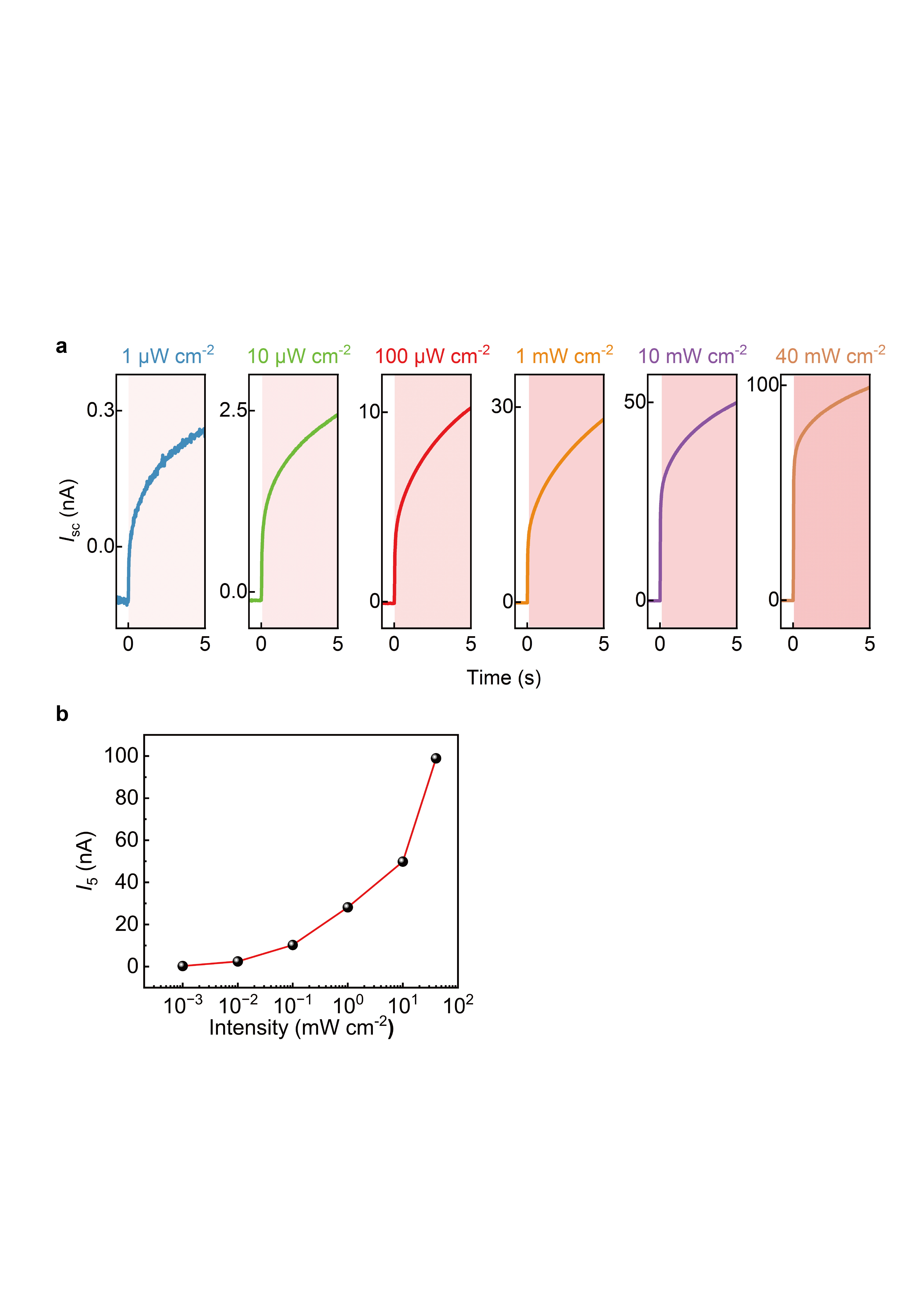
**

**Figure S12.** Dynamic photocurrent characteristics of ITO/MAPbI_3_/P3HT/Au memristor with P3HT thickness of 6.5 nm. a) *I*_sc_ versus illumination time curves under different light intensities (ranging from 1 μW cm^−2^ to 40 mW cm^−2^). b) Relationship between *I*_5_ and light intensity.

**
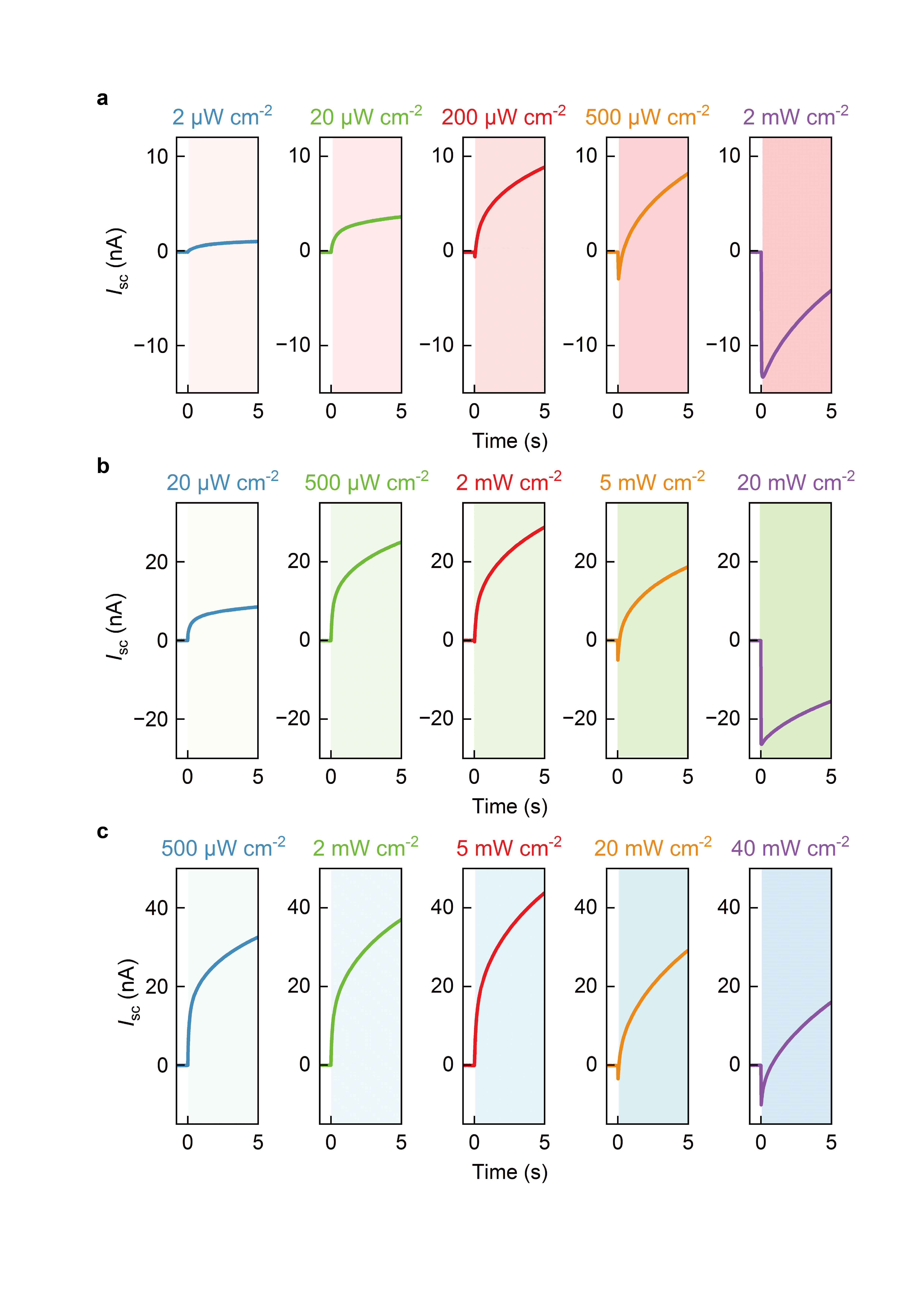
**

**Figure S13.** Effect of wavelengths on the dynamic photocurrent characteristics of ITO/MAPbI_3_/P3HT/Au memristor. a) red light, 626 nm. b) green light, 519 nm. c) blue light, 458 nm.

**

**

**Figure S14.** Detailed emission spectra of the used red, green and blue LEDs.





**Figure S15.** *I*_i_ and *I*_5_ as a function of light intensity.

**
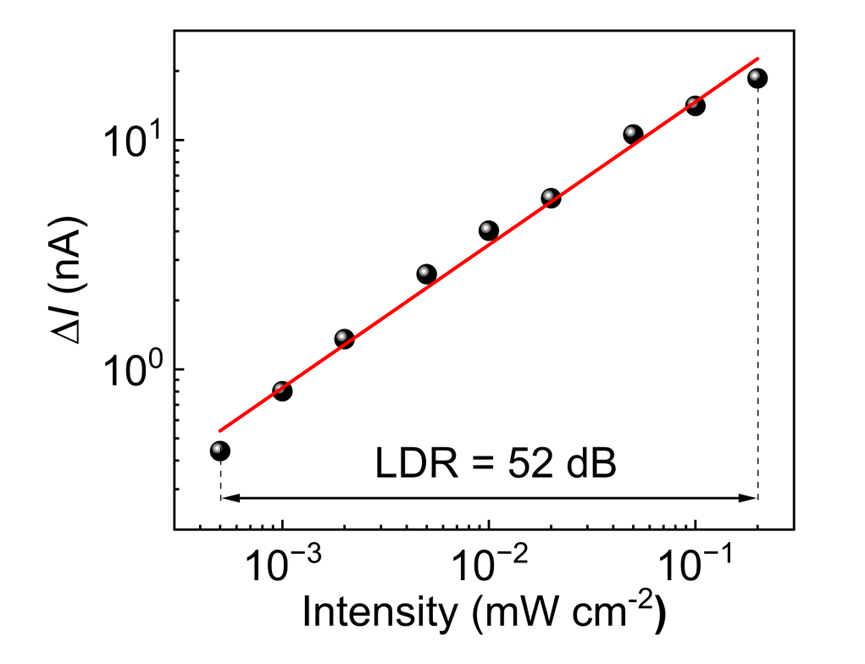
**

**Figure S16.** Relationship between Δ*I* and *L* on a log-log scale.

**
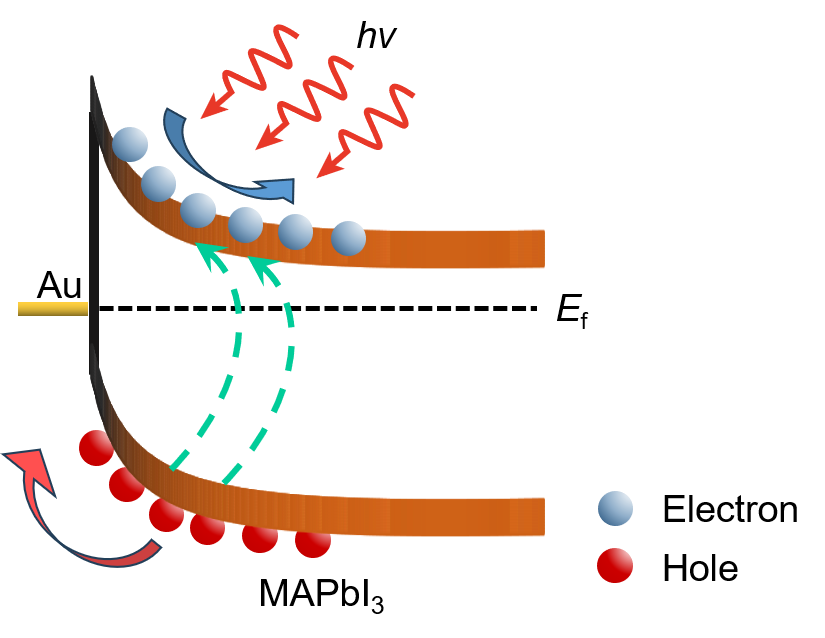
**

**Figure S17.** Working mechanism of ITO/MAPbI_3_/Au device under illumination. The Schottky junction at Au/n-type MAPbI_3_ interface dominates the photovoltaic direction.

**
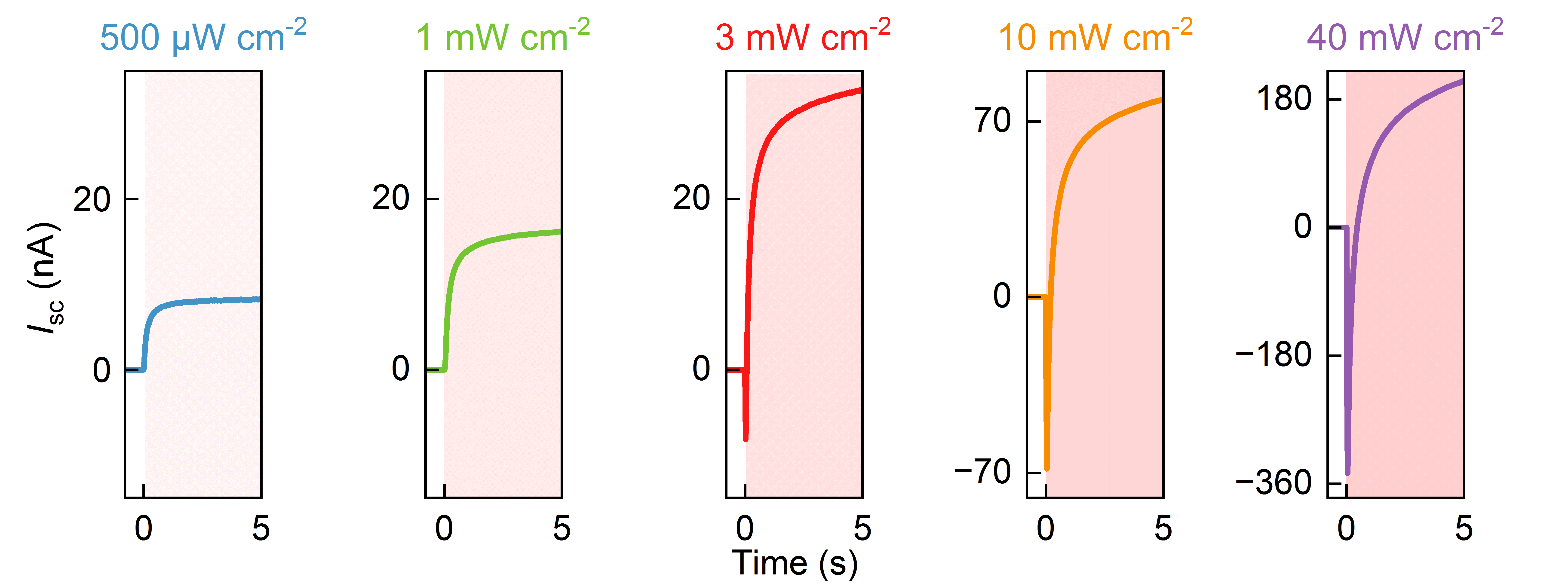
**

**Figure S18.** Real-time photoresponse (*I*_sc_) of ITO/MAPbI_3_/P3HT/ITO memristor under illumination with varied light intensities (5 s, 626 nm wavelength).

**
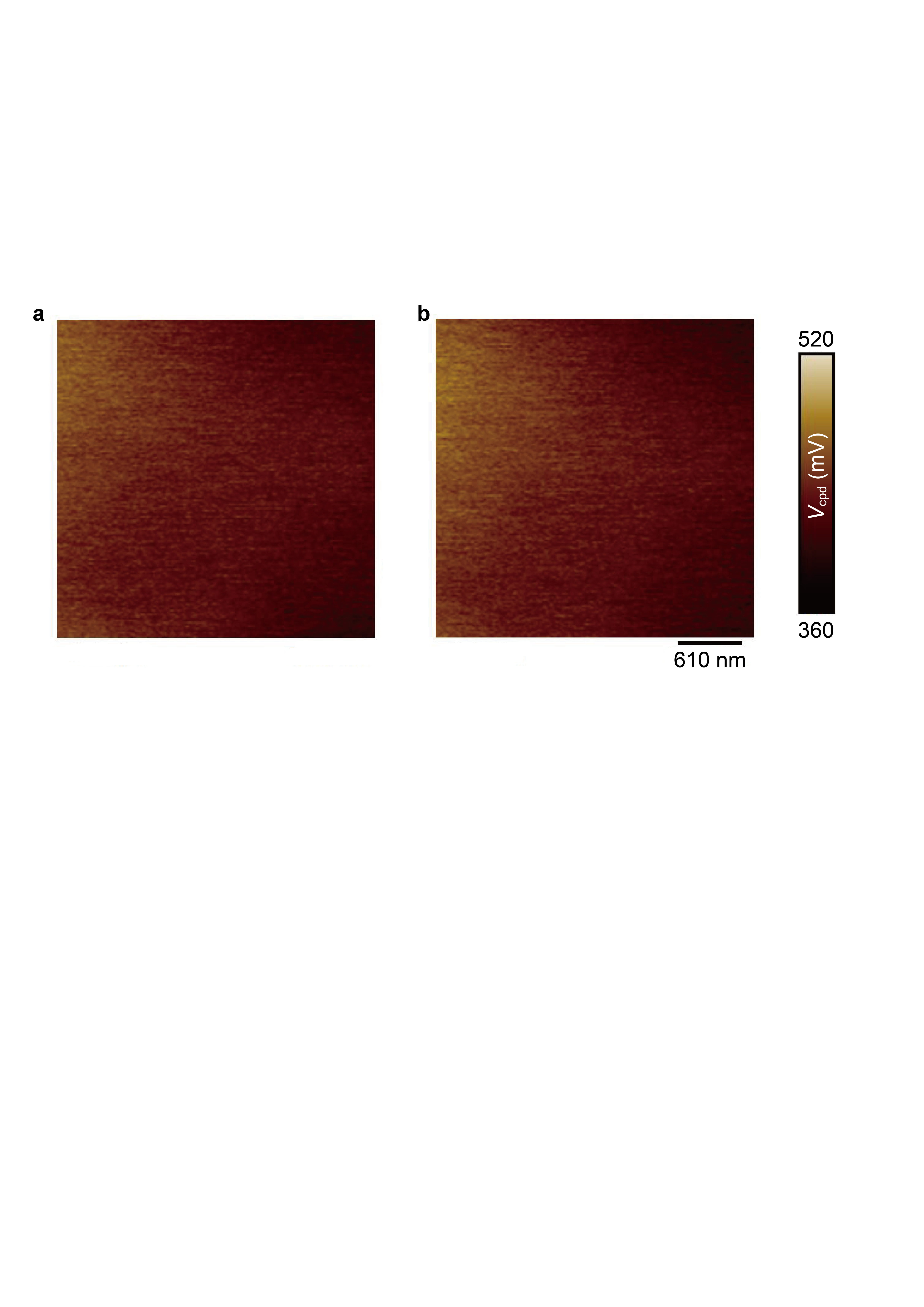
**

**Figure S19.** Recovery characteristics of surface potential of MAPbI_3_/P3HT device. a) Initial state in the dark. b) 20 min after turning off the light irradiation (50 s, 1 mW cm^−2^). The *V*_cpd_ can finally relax to its initial value after turning off the light irradiation.

**
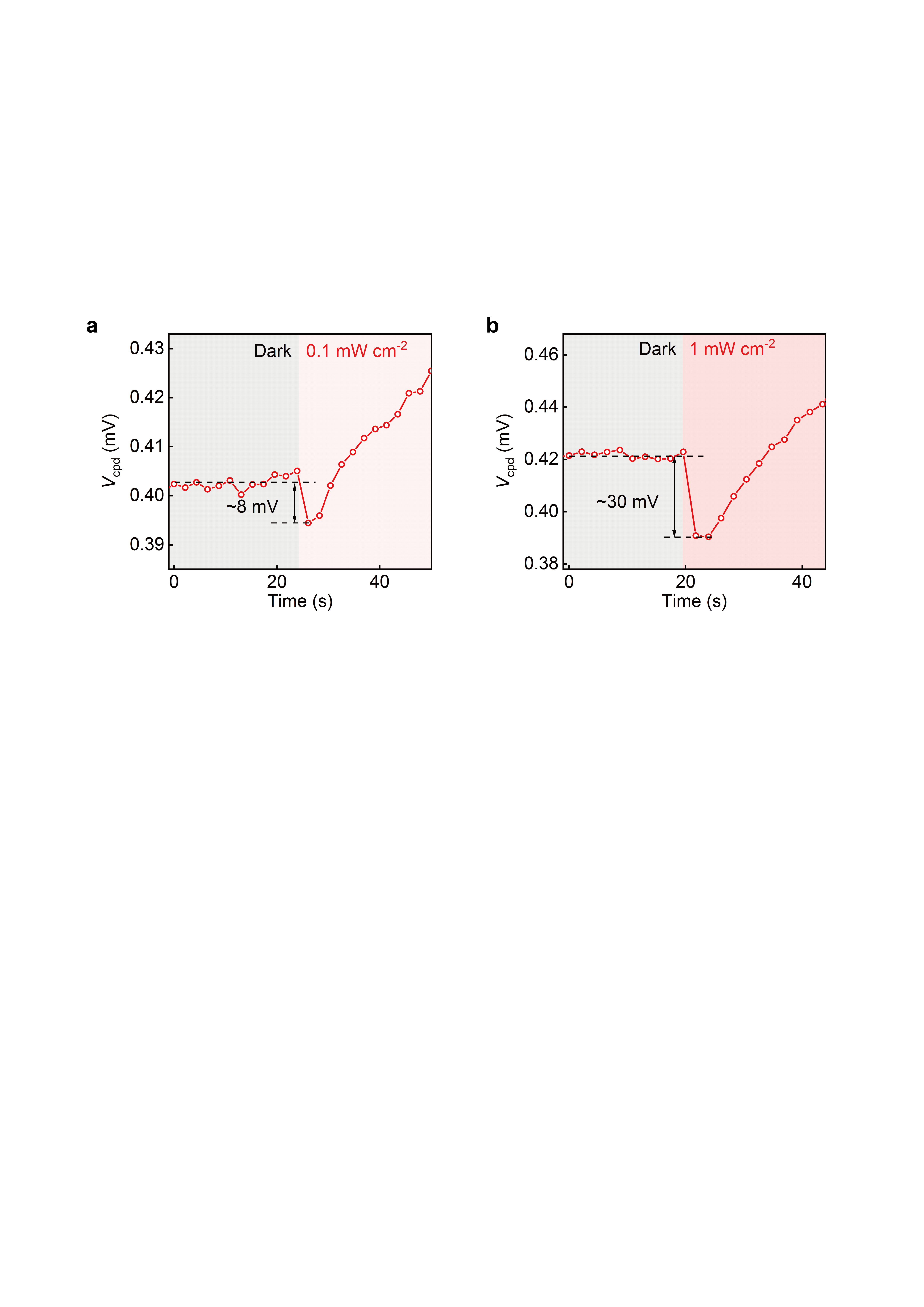
**

**Figure S20.** Real-time *V*_cpd_ change under illumination with varied light intensities. a) 0.1 mW cm^−2^. b) 1 mW cm^−2^. Increasing the light intensity from 0.1 to 1 mW cm^−2^ results in a significant decrease of transient *V*_cpd_ at the beginning of light irradiation with Δ*V*_cpd_ changing from 8 to 30 mV.

**
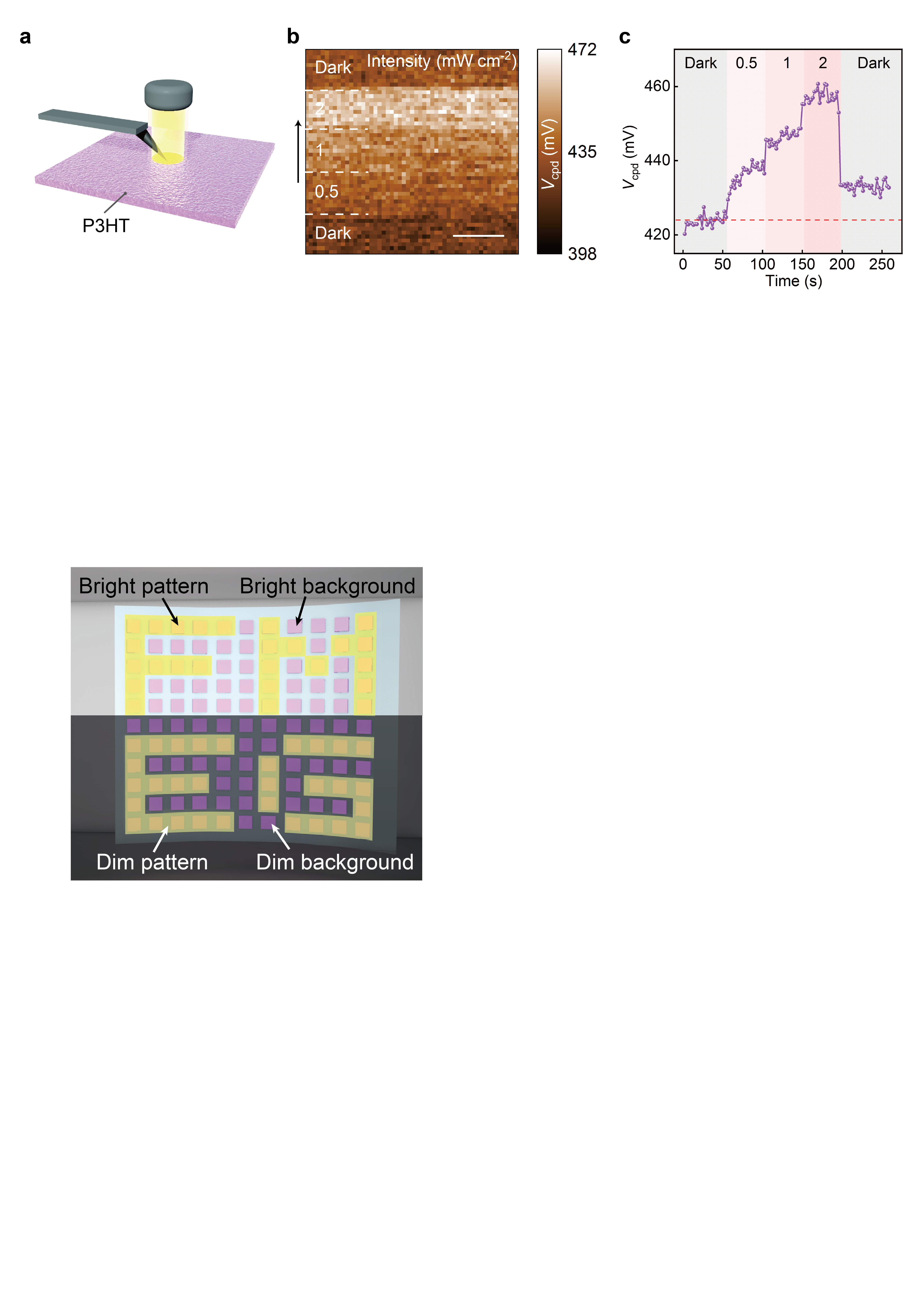
**

**Figure S21.** KPFM characterization of P3HT device. a) Schematic for KPFM characterization of P3HT device. b) Surface potential profile of P3HT device illuminated with a series of light intensity steps. Scale bar, 500 nm. The arrow indicates the time sequence of the test. c) Real-time *V*_cpd_ change extracted from b) Under light irradiation, the localized states in P3HT can trap photogenerated electrons, which results in the accumulation of holes and the increase of *V*_cpd_.

**
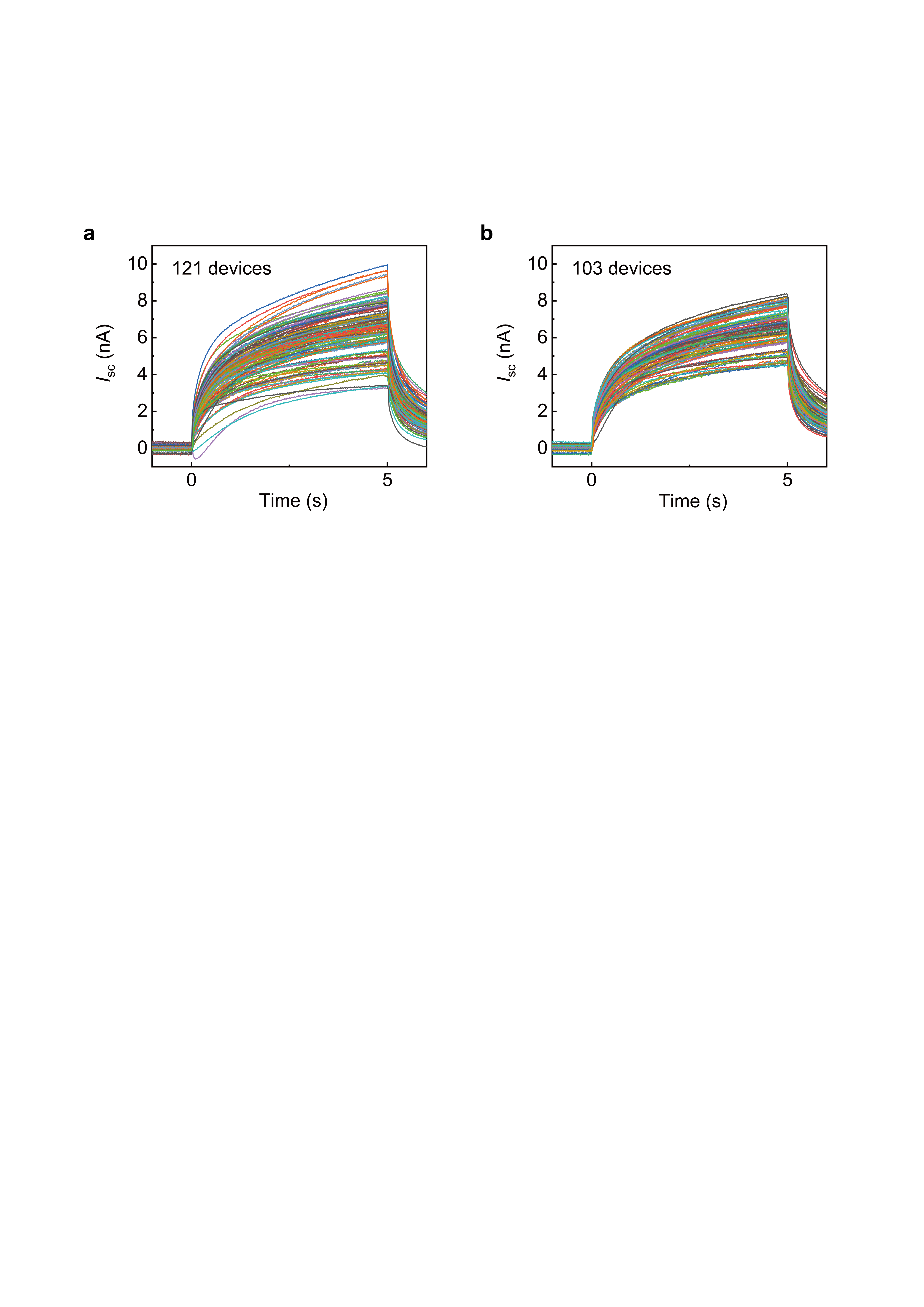
**

**Figure S22.** *I*_sc_ versus illumination time curves of all memristor pixels in an 11 × 11 crossbar array upon fixed light illumination (5 s, 626 nm wavelength, 20 μW cm^−2^). a) 121 devices. b) 103 devices. Among the 121 devices, 103 devices (85.12%) show a narrow photocurrent (*I*_5_) distribution between 4.6 and 8.4 nA.

**
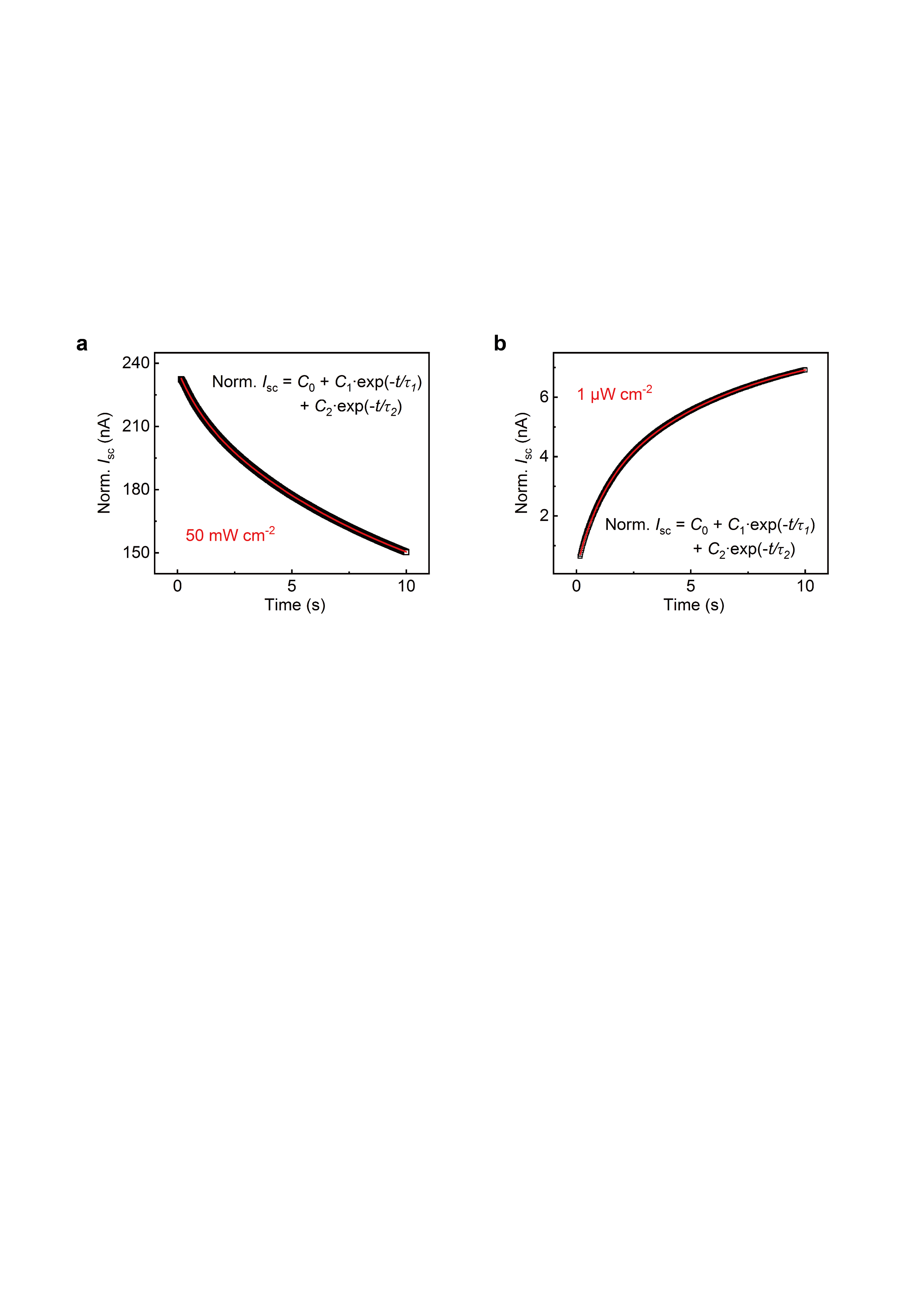
**

**Figure S23.** The average *I*_sc_ change over time under different light intensities. a) 50 mW cm^−2^. b) 1 μW cm^−2^. The average *I*_sc_ value can be well fitted by a double exponential decay function (red line).

**
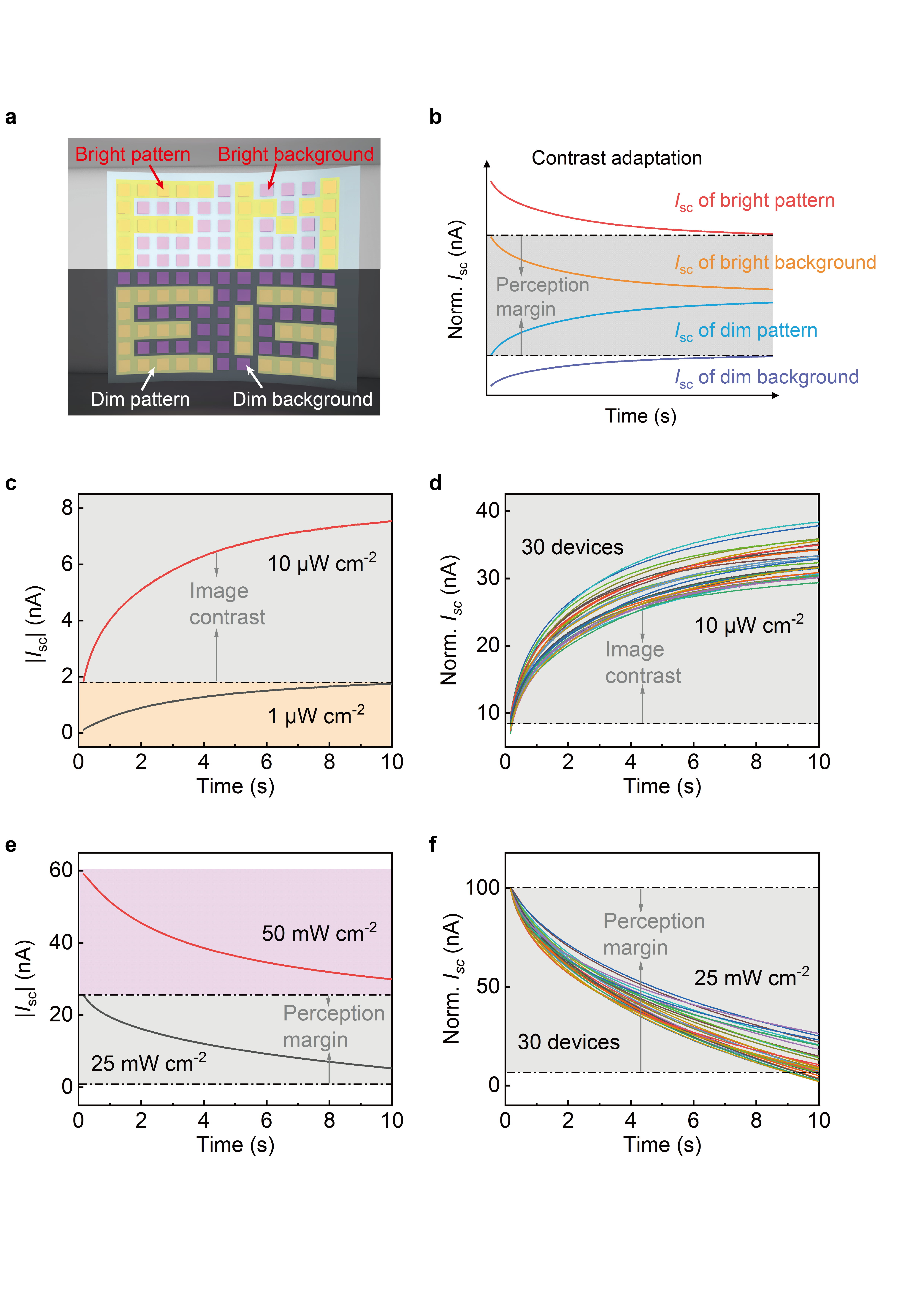
**

**Figure S24.** Mimicking active contrast adaptation functionality with memristor arrays. a) Schematic of an 11 × 11 memristor array to encode a high-contrast ‘FMEG’ pattern (94 dB). The character ‘EG’ (10 μW cm^−2^) in the lower half is under a dim-light background (1 μW cm^−2^), and the character ‘FM’ (50 mW cm^−2^) in the upper half is under a bright-light background (25 mW cm^−2^). b) Typical active contrast adaptation curves of the memristor array. c) *I*_sc_ versus illumination time curves for the shadowed ‘EG’ pattern before normalization (10 s, 519 nm wavelength). d) Normalized *I*_sc_ change over time in 30 devices corresponding to the character ‘EG’ (10 μW cm^−2^). e) *I*_sc_ versus illumination time curves for the highlighted ‘FM’ pattern before normalization. f) Normalized *I*_sc_ change over time in 30 devices corresponding to the bright-light background (25 mW cm^−2^).

**
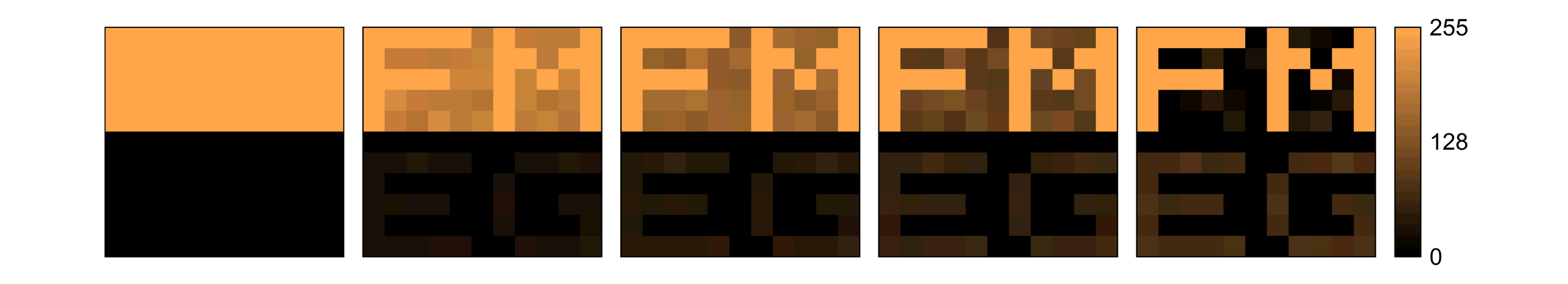
**

**Figure S25.** Time course of active contrast adaptation for the high-contrast ‘FMEG’ image in a grey level between 0 and 255.

To quantitatively evaluate the image contrast, the photocurrent value in **Figure 5d** was converted to a grey level between 0 and 255. The conversion formula is as follows:

$$G=\left\{ \begin{aligned} 0, &I_{\mathrm{sc}}\leq7 \\ \frac{I_{\mathrm{sc}}-7}{100-7}\times255, &{7<I}_{\mathrm{sc}}<100 \\ 255, &I_{\mathrm{sc}}>100 \end{aligned} \right.$$

where *G*­ is the grayscale value, *I*_sc_ is the normalized photocurrent value in **Figure 5d**.

**
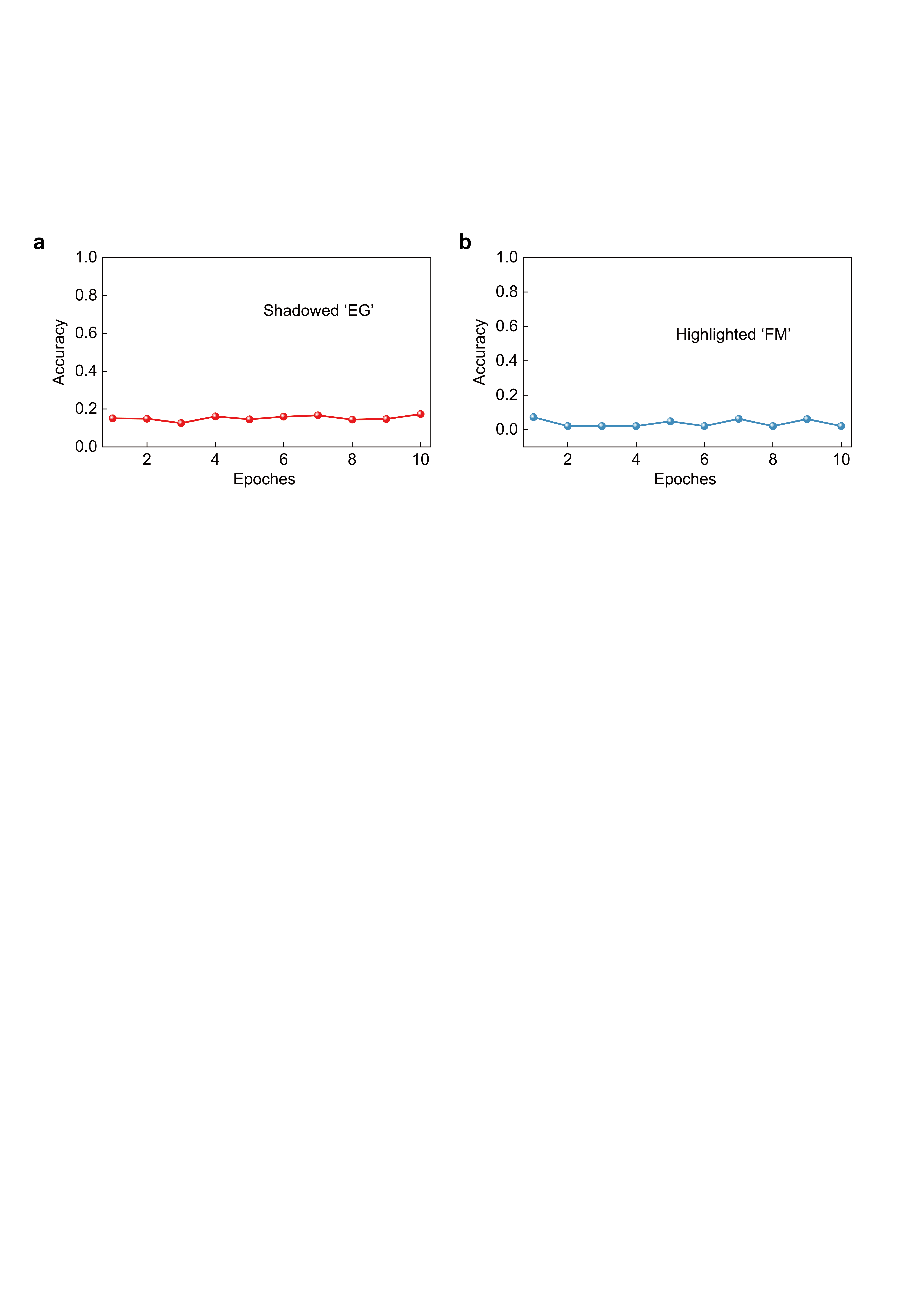
**

**Figure S26.** Recognition accuracy of the visual system with conventional image sensors. a) The shadowed ‘EG’ pattern. b) The highlighted ‘FM’ pattern.

**
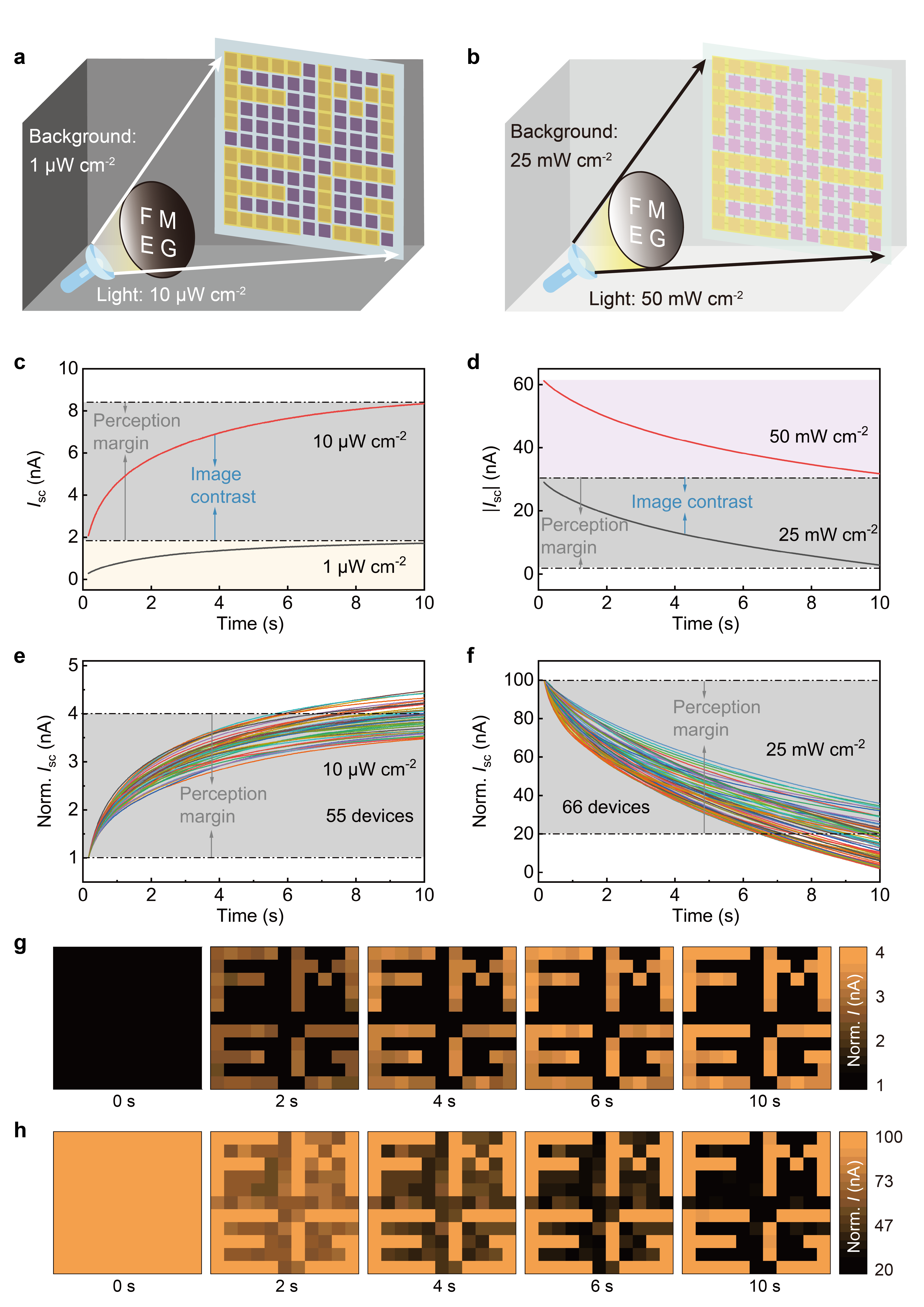
**

**Figure S27.** Mimicking scotopic and photopic adaptation functionality with memristor arrays. a) Schematic of an 11 × 11 memristor array under a dim background (1 μW cm^−2^) to encode a low-intensity (10 μW cm^−2^) pattern for the scotopic adaptation test. b) Schematic of an 11 × 11 memristor array under a bright background (25 mW cm^−2^) to encode a high-intensity (50 mW cm^−2^) pattern for the photopic adaptation test. c) *I*_sc_ versus illumination time curves under 1 μW cm^−2^ and 10 μW cm^−2^ before normalization. d) *I*_sc_ versus illumination time curves under 25 mW cm^−2^ and 50 mW cm^−2^ before normalization. e) Normalized *I*_sc_ change over time in 55 devices corresponding to the character ‘FMEG’ (10 μW cm^−2^). f) Normalized *I*_sc_ change over time in 66 devices corresponding to the bright background (25 mW cm^−2^). g,h) Temporal evolution of the ‘FMEG’ image during scotopic g) and photopic h) adaptation.

**

**

**Figure S28.** Effect of scotopic and photopic adaptation on improving pattern recognition. a) Illustration of a machine vision system based on a memristor array for visual adaptation and an ANN for pattern recognition. b) Top: temporal evolution of the EMNIST image ‘H’ during scotopic adaptation. Bottom: recognition accuracy of the visual system as a function of time for scotopic adaptation. c) Top: temporal evolution of the EMNIST image ‘H’ during photopic adaptation. Bottom: recognition accuracy of the visual system as a function of time for photopic adaptation.

**Table S1. Performance of the memristor under illumination with varied wavelengths.**

|  | *I*_ph_  (nA) | *R*_λ_  (A W^−1^) | *D*^*^  (cm Hz^1/2^ W^−1^) | NEP  (pW Hz^−1/2^) |
| --- | --- | --- | --- | --- |
| R (626 nm) | 0.56 | 5.6 | 1.87 × 10^13^ | 5.35 × 10^-4^ |
| G (519 nm) | 1.36 | 13.6 | 4.53 × 10^13^ | 2.21 × 10^-4^ |
| B (458 nm) | 2.86 | 28.6 | 9.53 × 10^13^ | 1.05 × 10^-4^ |

The responsivity (*R*_λ_),^[1]^ spectral detectivity (*D**)^[2]^ and noise equivalent power (NEP)^[3]^ can be expressed as follows:

$R_{\lambda}=\frac{I_{ph}}{L \times A}$ (1)

$D^{*}= \frac{R_{\lambda}A^{1/2}}{\left( 2eI_{dark} \right)^{1/2}}$ (2)

$\mathrm{NEP}= \frac{A^{1/2}}{D^{*}}$ (3)

where *I*_ph_ = *I*_5_ is the peak photocurrent, *L* = 1 μW cm^−2^ is the light intensity, *A* = 10^−4^ cm^2^ is the active area of the device, *e* is the electron charge, and *I*_dark_ = 2.8 × 10^−11^ A is the dark current. Detailed performance parameters of memristors under illumination with varied wavelengths (red light, 626 nm; green light, 519 nm and blue light, 458 nm) are listed in **Table S1** (Supporting Information). The highest detectivity of the device for 458 nm blue light is calculated to be 9.53 × 10^13^ cm Hz^1/2^ W^−1^.

**Table S2. The training and testing procedures of ANN.**

| **Step** | **Dataset** | **Environment** | **Target** | **Algorithm** |
| --- | --- | --- | --- | --- |
| 1 | Training set | Normal | Training network | Back propagation |
| 2 | Testing set | Normal | Verify accuracy |  |
| 3 | Testing set | High-contrast | Contrast adaptation |  |
| 4 | Testing set | Dim | Scotopic adaptation |  |
| 5 | Testing set | Bright | Photopic adaptation |  |

**Table S3. Comparison of the bioinspired active contrast adaptation memristor with previously reported visual adaptation devices.**

| Device structure | Mechanism | Visual adaptation | Scale | Energy  consumption (μJ)^b)^ | Adaptation  time (s) | Ref. |
| --- | --- | --- | --- | --- | --- | --- |
| A phototransistor based on  CsPb(Br_1-x_I_x_)_3_-MoS_2_ hybrid  Structure | Halide phase  segregation | Photopic adaptation | One  pixel | >4.2 | > 60 | [4] |
| A MoS_2_ phototransistor +  CsPbBr_3_-quantum-dots | Charge trapping/ detrapping | Photopic adaptation | One  pixel | >0.1 | > 2 | [5] |
| A CsFAMA^a)^ photovoltaic  device | Photovoltaic effect,  Ion migration | Photopic adaptation | One  pixel | >10^-4^ | 4.8 | [6] |
| An organic transistor that  consists of two bulk  heterojunctions | Photovoltaic effect,  Charge trapping/ detrapping | Photopic adaptation | 3 × 3 | 0.4 | 2 | [7] |
| A photovoltaic divider  and an ionotronic  synaptic transistor | Photovoltaic effect,  Ion migration | Scotopic adaptation,  Photopic adaptation | 3 × 3 | >1,  >0.1 | 10,  10 | [8] |
| A MoS_2_ phototransistor + ultraviolet/ozone treatment | Charge trapping/ detrapping | Scotopic adaptation,  Photopic adaptation | 8 × 8 | >456,  >21960 | 10,  80 | [9] |
| A phototransistor based on  Graphene/PbS-quantum-dots /graphene structure | Charge trapping/ detrapping | Scotopic adaptation,  Photopic adaptation | One  pixel | >30,  >120 | 100,  100 | [10] |
| An antagonistic photovoltaic memristor | Photovoltaic effect,  Photodoping, Charge trapping/detrapping | Scotopic adaptation,  Photopic adaptation,  Contrast adaptation | 11 × 11 | Self-powered | 2,  2,  1.14 | This work |
| ^a)^Ternary cation halide Cs_0.05_FA_0.81_MA_0.14_PbI_2.55_Br_0.45_; ^b)^The energy consumption of the device was calculated by *V* × *I* × *t*, where *V* is the program (electrical) or reading (optical) voltage, *I* is the current under *V*, *t* is the pulse width. | | | | | | |

**References**

[1] Y. Sun, L. Jiang, Z. Wang, Z. Hou, L. Dai, Y. Wang, J. Zhao, Y. Xie, L. Zhao, Z. Jiang, W. Ren, G. Niu, *ACS Nano* **2022**, 16, 20272-20280.

[2] Y. Yang, J. Liu, C. Zhao, Q. Liang, W. Dong, J. Shi, P. Wang, D. Kong, L. Lv, L. Jia, D. Wang, C. Huang, S. Zheng, M. Wang, F. Liu, P. Yu, J. Qiao, W. Ji, J. Zhou, *Adv. Mater.* **2024**, 36, 2307237.

[3] X. Li, M. Zhu, M. Du, Z. Lv, L. Zhang, Y. Li, Y. Yang, T. Yang, X. Li, K. Wang, H. Zhu, Y. Fang, *Small*, **2016**, 12, 595-601.

[4] S. Hong, S. H. Choi, J. Park, H. Yoo, J. Y. Oh, E. Hwang, D. H. Yoon, S. Kim, *ACS Nano* **2020**, 14, 9796-9806.

[5] D. Xie, L. Wei, M. Xie, L. Jiang, J. Yang, J. He, J. Jiang, *Adv. Funct. Mater.* **2021**, 31, 2010655.

[6] Q. Chen, Y. Zhang, S. Liu, T. Han, X. Chen, Y. Xu, Z. Meng, G. Zhang, X. Zheng, J. Zhao, G. Cao, G. Liu, *Adv. Intell. Syst.* **2020**, 2, 2000122.

[7] Z. He, H. Shen, D. Ye, L. Xiang, W. Zhao, J. Ding, F. Zhang, C. Di, D. Zhu, *Nat. Electron.* **2021**, 4, 522-529.

[8] S. M. Kwon, S. W. Cho, M. Kim, J. S. Heo, Y. H. Kim, S. K. Park, *Adv. Mater.* **2019**, 31, 1906433.

[9] F. Liao, Z. Zhou, B. J. Kim, J. Chen, J. Wang, T. Wan, Y. Zhou, A. T. Hoang, C. Wang, J. Kang, J. H. Ahn, Y. Chai, *Nat. Electron.* **2022**, 5, 84-91.

[10] M. Zhang, Z. Chi, G. Wang, Z. Fan, H. Wu, P. Yang, J. Yang, P. Yan, Z. Sun, *Adv. Mater.* **2022**, 34, 2205679.
